# The developmental effects of pentachlorophenol on zebrafish embryos during segmentation: A systematic view

**DOI:** 10.1038/srep25929

**Published:** 2016-05-16

**Authors:** Ting Xu, Jing Zhao, Zhifa Xu, Ruijie Pan, Daqiang Yin

**Affiliations:** 1 Post-doctoral Research Station of Civil Engineering, Tongji University, Shanghai 200092, China; 2 Key Laboratory of Yangtze River Water Environment, Ministry of Education, College of Environmental Science and Technology, Tongji University, Shanghai 200092, China; 3 Shanghai Collaborative Innovation Centre for WEEE Recycling, WEEE Research Center of Shanghai Polytechnic University, Shanghai 201209, China

## Abstract

Pentachlorophenol (PCP) is a typical toxicant and prevailing pollutant whose toxicity has been broadly investigated. However, previous studies did not specifically investigate the underlying mechanisms of its developmental toxicity. Here, we chose zebrafish embryos as the model, exposed them to 2 different concentrations of PCP, and sequenced their entire transcriptomes at 10 and 24 hours post-fertilization (hpf). The sequencing analysis revealed that high concentrations of PCP elicited systematic responses at both time points. By combining the enrichment terms with single genes, the results were further analyzed using three categories: metabolism, transporters, and organogenesis. Hyperactive glycolysis was the most outstanding feature of the transcriptome at 10 hpf. The entire system seemed to be hypoxic, although hypoxia-inducible factor-1α (HIF1α) may have been suppressed by the upregulation of prolyl hydroxylase domain enzymes (PHDs). At 24 hpf, PCP primarily affected somitogenesis and lens formation probably resulting from the disruption of embryonic body plan at earlier stages. The proposed underlying toxicological mechanism of PCP was based on the crosstalk between each clue. Our study attempted to describe the developmental toxicity of environmental pollutants from a systematic view. Meanwhile, some features of gene expression profiling could serve as markers of human health or ecological risk.

Environmental pollutants often inflict irreversible, long-lasting damage on animals during development^1^, and traditional toxicology does not provide sufficient information about the toxic mechanisms mainly due to limitations of the method. Pentachlorophenol (PCP), a typical uncoupler of oxidative phosphorylation (OXPHOS), is a representative environmental pollutant that was once a crucial pesticide and fungicide and is still ubiquitous in the environment in China^2^. Regardless of its relatively well-defined cytotoxic mechanism^3^,^4^, the impacts of PCP on animal development have remained obscure. Using a microarray, we found that PCP exposure activated glycolytic gene expression, forming a pattern similar to the Warburg effect and abnormally upregulated the embryonic cell cycle in zebrafish gastrula^5^. The effects on older embryos are the next logical question. In particular, segmentation is the stage after gastrulation during zebrafish development, and the first body morphology and movement appear in this period^6^. What are the impacts of PCP during this crucial stage? Will the disordered metabolic situation continue?

High-throughput technologies were expected to identify new aspects of the toxic mechanism. Compared with microarray, next-generation sequencing (NGS) is a better choice because it can detect novel sequences and provide a higher throughput^7^. Unfortunately, the massive data analysis following testing has been commonly believed to be a considerable challenge^8^. Theoretically, developing embryos/larvae could also be an optimal model for transcriptomic analysis because (1) they have simple but evolutionarily conserved structures, (2) they exhibit a dynamic response to a stimulus, and (3) they can provide accurate and generalizable information about the effects of chemical exposure on developmental processes^9^. A more attractive idea is that when a living body with a preliminary structure and function is exposed to pollutants, the effects could be easily traced back to and classified within a narrow range of origins. To accomplish this purpose, RNA sequencing is required to provide global information. This approach might simplify the abnormal organismal scenarios caused by the millions of toxic chemicals in environment and might be particularly beneficial for risk assessment and management.

In this study, we analyzed the transcriptomic effects of PCP treatments on zebrafish embryos using an Illumina HiSeq 2000 sequencing platform at 10 and 24 hours post-fertilization (hpf), representing the beginning and end of the zebrafish segmentation stage, respectively. The results suggested that PCP had significant impacts on multiple biological events in the developing embryos. Three different processes, metabolism, transmembrane transport and organogenesis, were chosen to ensure the integrity of causation. A mechanical framework that connected these events was proposed in an attempt to understand the occurrence and evolution of PCP’s developmental toxicity from a systematic view.

## Results

### Basic sequencing information

**Figure 1:**
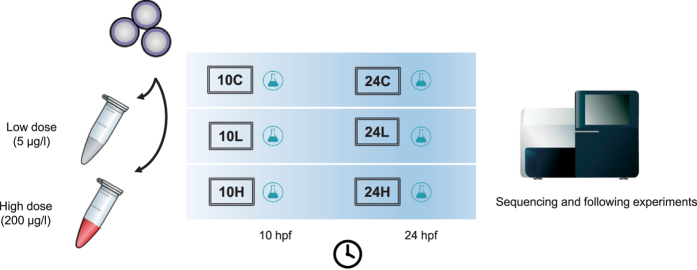
The diagram of exposure experimental design.

Our exposure experiments were illustrated as Fig. 1. After exposure, the sequencing data produced approximately 57–72 M total reads from 6 samples, and their mapping/unique mapping rates were all above 90%. The original RNA-Seq data are available in the Gene Expression Omnibus database (GSE75921). On most chromosomes, the distribution of the detected transcripts would not have suffered from PCP exposure, and only chromosomes 3, 6, and 19 varied in some extent (Fig. S1).

**Figure 2:**
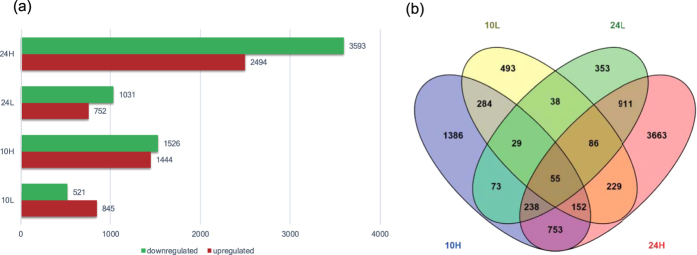
Differentially expressed genes in the four treatment groups. The genes were grouped based on the changes in their expression levels in response to different PCP concentrations and exposure durations. (**a**) Bar charts show the numbers of upregulated and downregulated genes. (**b**) The Venn diagram emphasizes the overlap between the significantly differentially expressed genes in the different groups.

The results of the differential expression analysis of the 21,227 detected transcripts are shown in Fig. 2a. A higher concentration and longer duration of exposure to PCP resulted in more differentially expressed genes, particularly those that were upregulated. Compared with the solvent control, 6,087 transcripts were affected in the 24H group (200 μg/l PCP at 24 hpf), of which 3,593 were upregulated and 2,494 were downregulated. The 10L group (5 μg/l PCP at 10 hpf) was the only treatment that included more upregulated genes than downregulated genes: 521 and 845 transcripts were up- and downregulated, respectively. The differentially expressed genes in the embryos treated with the two concentrations of PCP for two different durations are shown in a Venn diagram (Fig. 2b). A total of 5,895 genes were affected by only one of the four treatments: 3,663, 1,386, 493, and 353 genes, respectively. In comparison, only 55 genes were affected by all four treatments. We listed the top 10 upregulated mRNA transcripts in each of the 4 groups (Table 1) according to their fold changes (FC) and found that the genes in the two 10 hpf groups were completely different, while some of the genes in the two 24 hpf groups overlapped.

### Enrichment analysis based on the gene ontology (GO) definitions

Although the changes in gene expression seldom indicated an explicit biological impact on the organisms, a number of the enriched genes still provided useful clues that should not be neglected. Here, we performed a functional enrichment analysis of the genes based on the GO definitions using three domains: biological process (BP), molecular function (MF) and cellular component (CC). The complete list of the significant GO terms is shown in Supplementary Table 1–4, as there were too many entries to list here.

**Figure 3:**
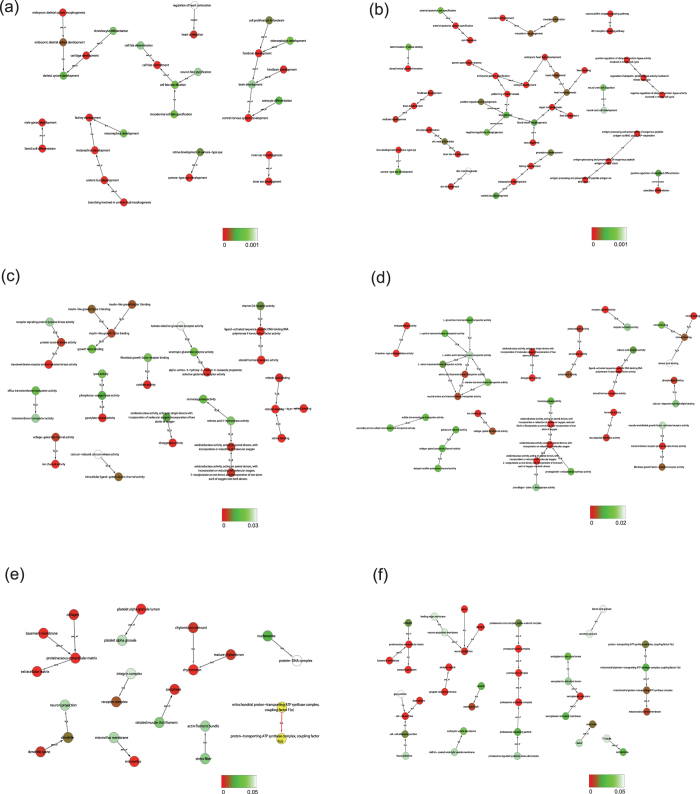
GO trees based on the hierarchies between the significant terms in the GO database. Only direct ancestors and child relationships were adopted, and the arrows point to the child terms. The colors of the balls represent the significance levels of terms. The six graphs include the BP terms in the (**a**) 10H group and (**b**) 24H group, the MF terms in the (**c**) 10H group and (**d**) 24H group, and the CC terms in the (**e**) 10H group and (**f**) 24H group.

To further filter the instructive terms from the abundant, affected terms, the GO trees of high-dose groups were graphed according to the hierarchical relationships of the terms, and only direct affiliations were accepted as valid (Fig. 3). If the toxic effects were primary and original, then we would expect their occurrence and development to be observed from a very early time point; therefore, they must continue to propagate and be the communal effects of the 10H and 24H groups. The BP terms tended to reflect PCP’s effects on tissue and organ development, such as the kidney, eye, heart (and vessels), and central nervous system. The MF terms highlighted internal binding and transporter activity. One notable class was the binding activities to retinoids, including retinol and its core metabolites, retinal and retinoic acid. The filtered components of the CC terms included extracellular matrix, neuron projection, and mitochondrion in both groups and cell junction and proteasome in the 24 H group.

### Enrichment analysis based on the Kyoto Encyclopedia of Genes and Genomes (KEGG) pathway definitions

**Figure 4:**
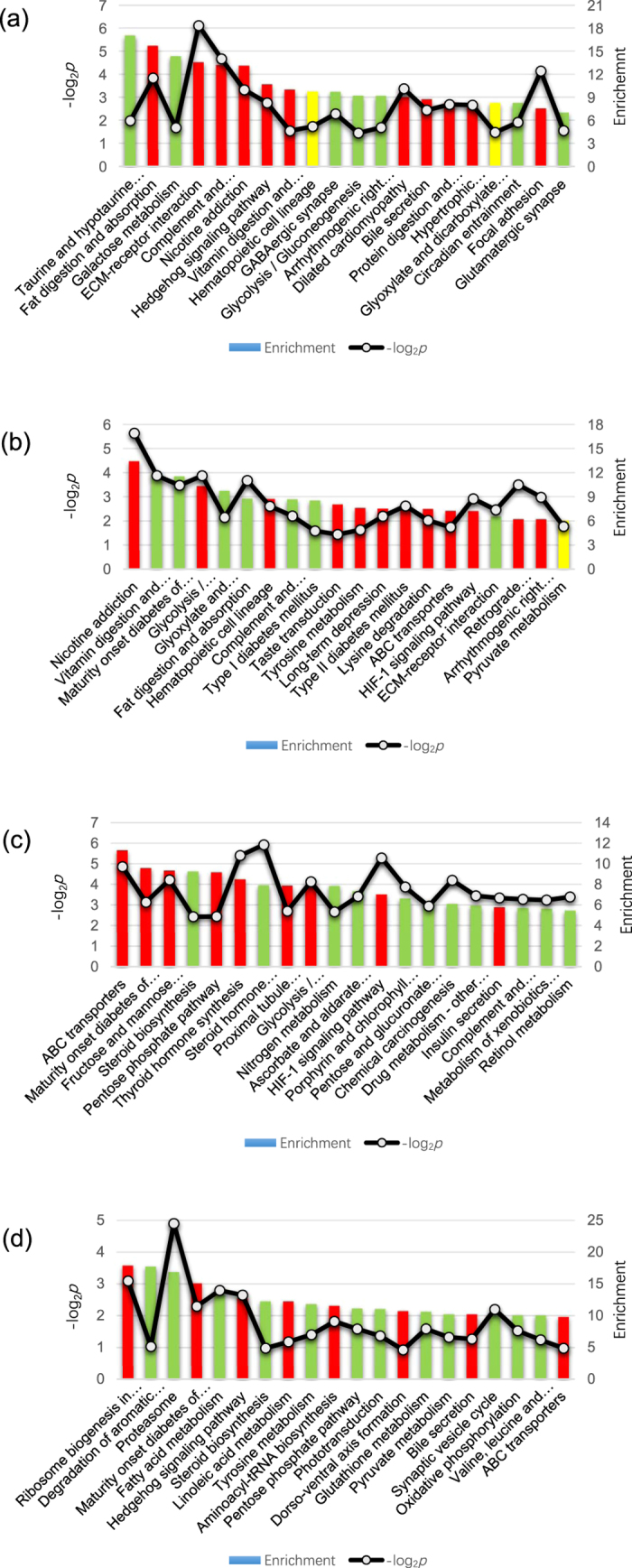
Top 20 enriched KEGG pathway terms of the four PCP-treated groups. The columns are graphed based on their enrichment levels, and the lines represent the *p*-values. (**a**) 10L group. (**b**) 10H group. (**c**) 24L group. (**d**) 24H group.

The pathway analysis was an important addition to the GO analysis. Figure 4 describes the top 20 affected terms based on the definitions and annotations from the KEGG database, which were sorted by enrichment level. The characteristics of the KEGG definitions highlighted metabolism-related pathways, such as glycolysis and pyruvate metabolism. Moreover, some terms, such as *ABC transporter, HIF1 signaling pathway*, and *ECM-receptor interaction*, appeared frequently.

**Figure 5:**
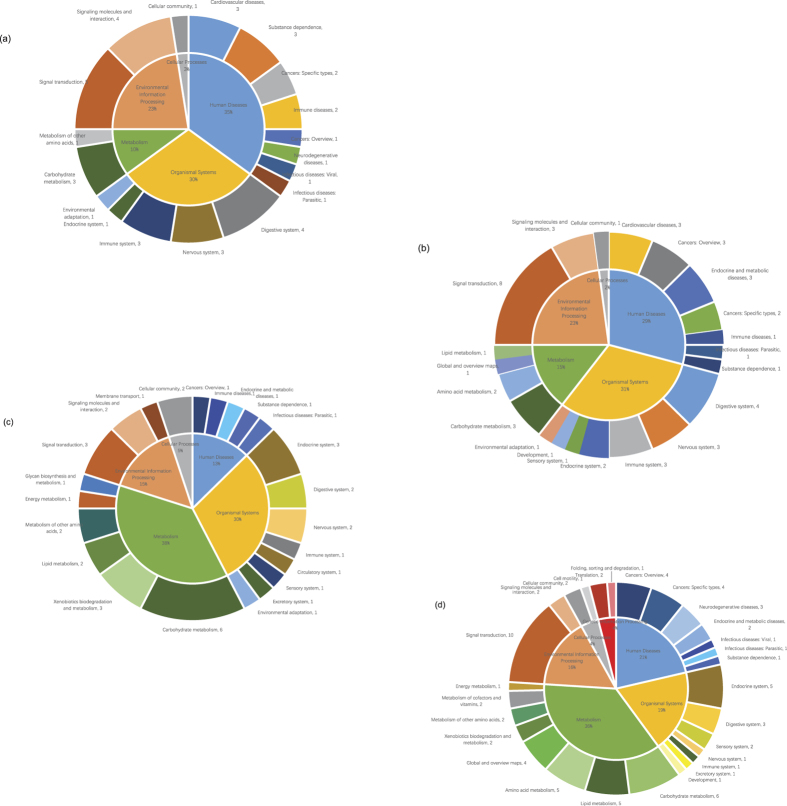
Proportions of individual categories of KEGG pathway terms. (**a**) 10L group. (**b**) 10H group. (**c**) 24L group. (**d**) 24H group.

The pie diagrams illustrate the relative proportions of the different types of terms (Fig. 5). The shares of the metabolic pathways varied from 7% to 38% in the four groups, corroborating a strong influence of PCP on embryonic metabolism. Among them, carbohydrate metabolism and its child term, glycolysis, were notable metabolic terms, which is consistent with the fact that many glycolytic genes exhibit tremendous fold changes. Many other downstream pathways involving lipids, amino acids, and other cofactors were also significantly regulated by exposure at 24 hpf likely resulting from alterations of the intermediates of carbohydrate metabolism. The digestive system terms, such as *fat digestion and absorption* and *vitamin digestion and absorption*, also had close connections with the metabolism terms.

### A mechanical framework for the developmental toxicity of PCP

**Figure 6:**
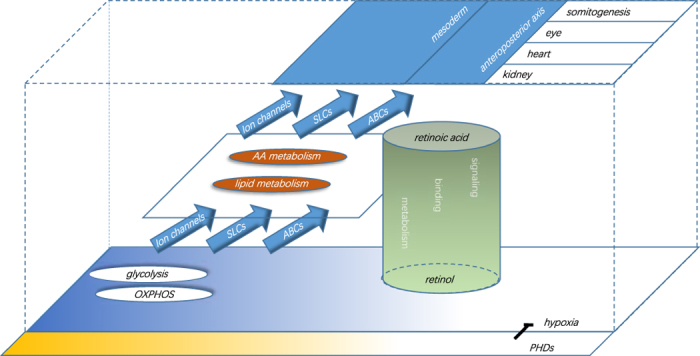
A proposed mechanical framework for PCP developmental toxicity. The vertical direction represents the progression of the toxic effects along with the causal biological events. The horizontal direction represents the approximate timeline of embryonic development and chemical exposure. The abbreviation “AA” in framework indicated amino acid.

The proposed toxicological process of PCP is shown in Fig. 6. We assumed that all of the effects of pollutant exposure may have had a common origin and evolved gradually during development from a specific early stage (for example, the zygote stage). Because numerous reports have demonstrated that there are close and mutual interactions between the assorted biological events that were influenced in our experiments, some of the significantly altered processes were selected and connected based on their established interactions. Energy metabolism was regarded as the origin of the effects because their outcomes were closely related to the physiological state of the organism. Somitogenesis and eye formation were the major upregulated and downregulated features at 24 hpf, respectively, regardless of the PCP dosage. The transmembrane processes most likely acted as intermediaries between different events. See the Discussion section for a more detailed description.

### Validation of the sequencing data

Because only approximately 5.6% and 4.0% of differentially expressed genes in the 10 hpf and 24 hpf embryos, respectively, were affected by low doses of PCP, qRT-PCR was performed on embryos that were exposed to high doses of PCP to validate the sequencing data at these two time points. The selected genes are listed in Table 2 and include *gapdhs, ldha* (for glycolysis), *atp5ia, cox7a2* (for oxidative phosphorylation), *egln3* (for the hypoxia response), *abcb11a, abcc8* (for ABC transporter), *mespa, her1, cryba2b* (for morphogenesis), *kcnc3b, scn1ba* (for ion channel), and *gnb5b* (for other functions). The definition of “significant” was only validated when *p* < 0.05 and |FC| > 2 were met simultaneously, thus aligning with the condition determined by sequencing. Using PCR amplification and quantification, the results confirmed that these genes had expression levels similar to those observed in the RNA sequencing.

### Determination of PCP concentration

A previous study showed that the actual toxicant concentrations decreased sharply upon exposure in a polystyrene microplate^10^. Hence, the actual nominal concentration of an initial 200 μg/l PCP dose was determined at the beginning and end of the exposure period using chromatography (Table 3). The measured concentration at the end of the exposure was 112.57 ± 2.95 μg/l, which was slightly higher than the half-nominal concentration. This actual value could be observed in extreme conditions in the actual environment after degradation/absorption^2^, indicating that our study had some ecological significance.

## Discussion

The affected processes used to construct a mechanical framework should meet the following requirements: (1) they are supported by some relevant and statistically significant terms at both time points and (2) the involved significant genes or gene families are necessary for the biological process. Based on these criteria, metabolism, transmembrane transporter, and organogenesis were the main components of the framework.

### Affected energy metabolism-associated events

The role of energy is extremely fundamental to biological metabolism. Although OXPHOS provides approximately 90% of the energy, glycolysis has the advantage of generating energy rapidly and is efficient under some situations^11^. PCP exposure significantly affected glycolysis, OXPHOS, and their downstream carbohydrate, amino acid, and lipid metabolism pathways.

#### Glycolysis and hypoxia

The unusual activities of genes in the glycolysis and subsequent lactate fermentation pathways were the most remarkable characteristics of the zebrafish transcriptome, particularly in the 10H group, and were quite similar to those of the 8 hpf transcriptome in our previous study^5^. Both high-dose short-term and low-dose long-term exposure induced glycolysis activity but affected different genes. Most of the glycolytic genes, e.g., *gapdhs, eno1a, pfkfb3, aldocb*, and *ldha*, were highly active in the 10H group (Table S5) but were restored to their normal levels at 24 hpf. In contrast, some of the other glycolytic genes, e.g., *aldob, pfkma*, and *gpia*, were upregulated at 24 hpf. Two key genes that were treated as indicators of the Warburg effect, *ldha* and *pkma*, were only induced in the 10H group, suggesting that glycolysis may lead to potentially different consequences at the two stages.

Hypoxia and its core indicator, hypoxia-inducible factor-1α (HIF1α), were highly correlated with the glycolytic process. Even in aerobic glycolysis, in which the oxygen supply was adequate, HIF1α expression fluctuated abnormally^12^. In addition to the glycolytic genes, most of the top 10 upregulated genes in the 10H group, such as *egln3, egln2, slc2a3b*, and *igfbp1a*, were thought to respond to cellular hypoxia. For example, *igfbp1* overexpression was strongly induced by HIF1α and delayed zebrafish development^13^. Nevertheless, HIF1α expression itself remained fairly stable throughout the process, similar to that at 8 hpf^5^, and was even downregulated in the 24H group.

In light of the present knowledge, HIF1α may have been affected by the continuous, high expression of *egln3* and *egln2*. These two transcripts encoded prolyl hydroxylase domain enzymes (PHDs) 3 and 2, respectively, which could be induced by hypoxia and then destabilize and degrade the HIF1α protein^14^. The restricted HIF1α expression may protect the zebrafish embryos from severe hypoxia in this experiment, although the actual roles of PHDs remain controversial. However, a dilemma of why the HIF1α-induced genes could be activated in the absence of HIF1α activation emerged, and it lacked a convincing explanation.

#### OXPHOS

Traditionally, PCP has been recognized as a typical OXPHOS uncoupler. However, PCP exposure resulted in significant (24H) or mild (10H) inhibition of the embryonic OXPHOS, electron transport chain (ETC), and proton transport processes (Table S5). We noticed that in complex II, an alternative of complex I that shares part of the ETC and the citric acid cycle, two hydrophobic subunits, *sdhc* and *sdhd*, which transferred the electrons to ubiquinone (coenzyme Q10), were downregulated after exposure, while two hydrophilic subunits, *sdha* and *sdhb*, which have no Q10 pool, were unaffected. The extensive therapeutic roles of Q10 in cancer, male infertility, heart failure, and other pathologies have been highlighted recently^15^,^16^,^17^, findings that may indicate underlying correlations between metabolism and chemical toxicity/diseases. OXPHOS often opposes glycolysis and hypoxia/HIF1α, particularly in cancer studies^18^,^19^; however, in our experiments, they were not strongly correlated.

#### Retinoid metabolism

Similar to a “bagman”, retinoids exhibited complicated relations with energy metabolism and the subsequent metabolic events. The metabolism, binding and signaling of retinoids were crucial, particularly for facial and cephalic morphogenesis, visual perception, and somitogenesis^20^,^21^. Retinoic acid, a crucial maternal signal, was primarily regulated by retinaldehyde dehydrogenases (RALDHs) and one of the cytochrome P450 isoforms, CYP26A1^20^. As shown in Table S6, PCP disrupted retinoid binding at both 10 and 24 hpf. The affected families were retinol-binding proteins, cellular retinoic acid-binding proteins, RALDHs (at 10 & 24 hpf), RA receptors (RARs), and retinoid X receptors (RXRs) (at 24 hpf). *cyp26a1* upregulation (FC = 4.25 in the 24H group) echoed the alterations of the RARs/RXRs, suggesting that PCP has the potential to disrupt the entire retinoic acid signaling pathway.

### Changes in the embryonic electrophysiological transport processes

Taking humans as an example, approximately 10% of all genes are transporter related, which is consistent with their biological significance in maintaining the homeostasis of the organism^22^. Both the cargo they carried and the proteins themselves were useful for determining the underlying mechanism. In this test, the results of GO and KEGG pathway analyses highlighted the importance of the transmembrane transport processes in response to PCP exposure (Table S7).

#### Solute carrier family

Some members of the solute carrier (SLC) family were consistently altered from 10 to 24 hpf. Three members, *slc4a1a, slc16a9a* and *LOC100150452*, were the primary differentially expressed genes in the 10H group. The last two were upregulated and likely regulated carnitine and creatine transport^23^,^24^, both of which are critical metabolic agents that participate in ATP production and cellular energy homeostasis^25^. Slc4a1a encodes the Band 3 anion transport protein, the primary Cl-HCO_3_ exchanger that regulates intracellular pH and chloride levels and that is downregulated in various cancer tissues^26^. Slc38a3 exhibited the highest expression levels among the dozens of altered genes in the 24H group, and its increased expression correlated with potassium restriction and metabolic acidification^27^. A conspicuous cluster in the 24H group included three transcripts from the SLC25 family, *slc25a4, slc25a25a*, and *slc25a38a*. The SLC25 family is known to be associated with mitochondrial and cytosolic metabolism, along with subsequent physiological processes, such as OXPHOS and gluconeogenesis^28^. Among them, SLC25A4 and SLC25A25, which transport ATP and phosphate, respectively, were significantly inhibited by PCP treatment. Most of the remaining genes were involved in amino acid transport.

#### Ion channels and synaptic transmission

The role of ion channels in early vertebrate development has been broadly described. However, other studies have already demonstrated the crucial functions of ion channels and pumps in early embryonic morphogenesis and hatching^29^,^30^,^31^. Here, we observed that PCP treatment influenced the genes encoding cation channels, including potassium, sodium, and calcium channels. Most of these channels were voltage-gated channels, with the exception of the partial potassium channels, which are “inwardly rectifying channels”. Compared with the expression levels of the SLCs, the expression levels of the ion channels were relatively lower, except for *cacna1fb*, which was expressed at 10-fold higher levels than the other members. This gene participates in signal transmission in synaptogenesis and synaptic connectivity in the retina^32^ and skeletal muscle^33^. In contrast, *kcnk5* and *kcnh2* are mainly expressed in the kidney and heart, respectively^34^,^35^. PCP also disrupted two members of the degenerin/epithelial Na^+^ channel family, *accn1* and *accn5*; the latter is a bile acid-sensitive subunit^36^.

Two key functions of ion channels are to assemble and to regulate neurotransmitter (NT) receptors. Therefore, as expected, our enrichment analysis obtained various NT-related terms, including glutamatergic, serotonergic, dopaminergic, cholinergic, and GABAergic synapses. In addition to ion channels, AMPA receptors (AMPARs), a type of ionotropic glutamate receptor, were affected by PCP exposure. Physiological and pharmacological studies have demonstrated that AMPARs participate in the development of mammalian and zebrafish central nervous systems^37^. These features also suggested that the neurobehavioral effect could be regarded as a marker for PCP’s developmental toxicity.

#### ATP-binding cassette importers and exporters

The ATP-binding cassette (ABC) transporter family includes transmembrane proteins that pump exogenous toxicants and drugs out of the cell and that act as a barrier. In our test, many family members, including *abcb11a, abcg2a, abcg8, abcc12*, and the pathway term *ABC transporters* were the most significantly altered in the 24L group. Zebrafish lack the ABCB1/P-glycoprotein, the major xenobiotic efflux pump in human and rodents. Its physiological roles are executed by the homologs ABCB4 and ABCB5^38^, thus demonstrating that *abcb5* was upregulated and reflecting the stress due to PCP exposure. However, the most impressive representation was from *abcb11* (also known as BSEP/SPGP), which exhibited a 27.21-fold change in the 24H group. Although the existing research in human cell lines showed that ABCB11 was rarely involved in xenobiotic metabolism^39^, our result was consistent with the significant upregulation of the *abcb11* gene after perfluorooctane sulfonate exposure^40^, suggesting that this exporter of endogenous substances has a certain role in exogenous exposure. Likewise, abcg2/BCRP, which is a high-capacity urate exporter^41^, was essential for the disposition and excretion of xenobiotics in rainbow trout^42^.

### Effects on segmentation stages of zebrafish morphogenesis

Zebrafish embryos gradually become a complex organism after 14 h of segmentation stages mainly via the mesodermal differentiation and anteroposterior axis establishment. The rudiments of some primary organs form, including myotome/sclerotome (from somite), pronephros, neurula (which mature further into the brain, eye, and ear), and heart^6^. Although most of these tissues were influenced by PCP, two of the developmental events, somitogenesis and lens formation, were the most significant and, therefore, are discussed here.

#### Somitogenesis

Somitogenesis (the formation of somites from the mesoderm along the anteroposterior axis) is the most characteristic feature of the segmentation stage and was seriously affected by PCP (Table S8). The Mesp and Hairy protein families have central roles in this event^43^. Their members, including *mespaa, mespab, her1*, and *msgn1*, showed moderate changes at 10 hpf and vigorous upregulation at 24 hpf. The oscillations in the expression levels of these genes in the pre-somitic mesoderm, the so-called “Clock and Wavefront mechanism”^44^,^45^, were maintained during segmentation; however, in general, their expression levels exhibited a decreasing trend. Therefore, their upregulation implied a delay of developmental progress and likely affected the subsequent formation of bones and muscles. Because somitogenesis was accurately regulated by the competition between Wnt/Fgf signals and RA signals, the evidence supporting this hypothesis was that *wnt3a, fgf8a*, while *cyp26a1* (the gene encoding an enzyme to metabolize RA into bio-inactive form of retinoids) were upregulated.

#### Eye lens and other sensory organs

Zebrafish eyes were clearly recognizable in the embryonic head at 24 hpf, and the development of the visual system was likely considered a representative response to xenobiotics during early development. The most downregulated GO MF term in both 24 hpf groups was *structural constituent of eye lens*, composed of members encoding the crystalline-family proteins, which are the essential components of the eye lens. Crystallins could be classified into the α-, β-, and γ-subfamilies, and PCP primarily influenced the β- and γ-crystallins (Table S9). This phenomenon could be explained by the fact that α-crystallin accounts for less than 1% of the crystallins in larvae prior to 10 days post-fertilization (dpf)^46^. Meanwhile, the γM-crystallins, which are essential for underwater vision, were the most abundant subunits^47^. The embryonic ocular lens is responsible for focusing on objects at different distances and protecting the eye from ultraviolet light; it enlarges from 24 to 72 hpf in zebrafish^48^. Therefore, damage to the lens could likely encumber subsequent eye development.

Even as early as 10 hpf, the enrichment analysis identified abnormally expressed genes in the eye, the ear, and the olfactory system. Photoreceptor cell morphogenesis was interrupted by BDE47 in 6 dpf zebrafish larvae in our previous study^49^. These facts reminded us that environmental pollution can cause remarkable damage to the eyes and other sensory organs of fish. The senses, particularly vision, guide most larval and adult behaviors; therefore, their impaired functions may have profound impacts on fish survival and reproduction in real ecosystems.

### Systematic views of developmental toxicity

Explaining the massive data obtained from the high-throughput tests has become the greatest bottleneck in -omics fields. It is impossible for all of the existing enrichment terminologies to connect cross-category events, although inextricable links, such as glycolysis and hypoxia, have been identified between them. This reason might contribute to why the action mechanism of pollutants could not be clarified by enrichment analysis alone and why we proposed a hypothetical framework to understand the toxic mechanism.

A basic setting for the framework is that the biological events that are most heavily affected are also indicators for PCP exposure. From a systematic viewpoint, we tended to focus on embryonic toxicological phenomena that are derived from abnormal energy metabolism, because of their consistent performance during 8–10 hpf and because of their profound consequences. In addition to the ATP outcomes, OXPHOS influenced the cellular membrane potential, and glycolysis affected other downstream metabolic pathways through lactate/pyruvate and altered cellular acidity. These metabolic events also had inherent connections with cellular oxygen and hypoxia. The changes in glycolysis/carbohydrate metabolism have also been observed in large toxicological studies^50^,^51^,^52^ but have rarely been investigated. The subsequent physiological conditions further influenced transmembrane transport. pH controls, enhances or inhibits many members of SLC and ABC transporter families, and ATP/ADP modulates the K currents from the voltage-gated potassium channels^53^. The metabolic products of glycolysis are known to be essential for subsequent metabolic pathways, such as lipid and amino acid metabolism.

At 24 hpf, the embryos have sufficiently matured to display morphological features. Thus, the toxic effects on organ formation could be the extrinsic and terminal parts of the framework. Moreover, the impacts of PCP on zebrafish organogenesis seemed to be associated with retinoid actions. Retinoids have two main active forms: all-*trans* retinoic acid and 11-*cis* retinal. Given the roles of retinoic acid signaling in somitogenesis (antagonist) and eye development (agonist)^54^,^55^, PCP was suggested to influence these two processes by repressing RA signaling. RA signaling has been shown to regulate the formation of the mesoderm and anteroposterior axis, and retinoid metabolism has been associated with other metabolic pathways. Previous studies have described possible interactions between retinoids and glycolysis/hypoxia, and other studies may be ongoing.

Here, we proposed a framework to study the mechanism of xenobiotic exposure from a systematic and progressive viewpoint. The framework connected disparate biological events, particularly physiological events (metabolism), to gene regulation events (organogenesis) to convert the disorganized transcription information into a readable mechanical story. If we attained our goal, we expected to identify common parts in the toxic mechanisms of millions of chemicals. However, our present study was challenging, and more details must be collected to fill in the gaps in this framework.

## Methods

### Zebrafish and toxicological exposure

Wild-type Tuebingen zebrafish (*Danio rerio*) were maintained in a recirculating filtration system using water treated by reverse osmosis (ESEN, China). The protocols for zebrafish feeding and spawning and water quality monitoring have been previously reported^49^. PCP (purity >98%, Sigma, New Haven, CT) was dissolved in dimethyl sulfoxide (DMSO) as the vehicle (purity >98%, Amresco, Solon, OH). The final concentrations of PCP were 0, 5, and 200 μg/l, each of which contained 0.01% vehicle. The exposure started at approximately 1 hpf in a 28.5 °C incubator and was stopped at 10 and 24 hpf, respectively. Approximately sixty embryos in each treatment group were used for deep sequencing. For the quantitative PCR assay, the PCP treatments and DMSO controls were replicated 3 times. All animal protocols were carried out in accordance with guidelines for care and use of laboratory animals as approved by the Animal Ethics Committee of Tongji University.

### Chemical determination

To investigate the influence of the exposure duration on the PCP concentration, the actual concentrations of a nominal 200 μg/l PCP solution were determined at the beginning and end of the exposure using an Agilent 1200 liquid chromatography system (Waldbronn, Germany) with a VW detector operating at a wavelength of 305 nm. The constituents of the mobile phase were methanol and 1 g/l ammonium acetate (V/V: 70/30), with a flow rate of 1 ml/min. The injection volume was 20 μl. A TC-C18 reversed-phase column (150 mm × 4.6 mm, Agilent Technologies, Palo Alto, CA, USA) was used, and the column temperature was set to 25 °C. The separation was completed in 8 min.

### RNA sequencing experiments, differential gene expression analysis and functional enrichment analysis

The experimental and analysis protocols have been previously reported^56^. The total RNA was extracted and purified using an RNeasy Mini Kit (Qiagen, Germany) according to the manufacturers’ instructions. The RNA library was constructed, and sequencing was performed using an Illumina HiSeq 2000 platform (San Diego, CA, USA) after the RNA quality was examined using an Agilent TapeStation 2200 (Santa Clara, CA, USA). The clean reads from the raw sequencing data were mapped into the reference zebrafish genome Zv10 assembly using Tophat package v2.0, and further into human GRCH37.p13 genome by BLAST algorithm to achieve sufficient annotations. The mapped data were then normalized using the trimmed mean of the M-values (TMM) in edgeR package, and TMM counts over 0 were defined as detected. The statistical analysis for differential expression was performed using the R language tool of DEGseq, and “significance” was validated when *p* < 0.05 and |FC| > 2. To identify the statistical significance of the differentially expressed genes between the control and treatment groups, the gene functional enrichment analysis was then analyzed using Fisher’s exact test and the definitions and annotations of the GO terminology and KEGG pathway databases. The significance threshold was set as *p* < 0.05, with the exception of the GO BP terms, which used *p* < 0.001. The GO trees were illustrated based on the hierarchies between the significant terms using Cytoscape 3.2.1, and only a direct relationship between the ancestors and children was adopted.

### Quantitative real-time PCR and data analysis

After quality control of the extraction, the total RNA was reverse-transcribed into cDNAs with Superscript III Reverse Transcriptase (Invitrogen), random hexamers and oligo(dT) primers. SYBR Green I Quantitative PCR was performed on a 7500 Fast Real-Time PCR system (Applied Biosystems, Foster City, CA, USA). The PCR amplification mixture consisted of a 20-μl final volume containing 0.2 μl of Platinum Taq Polymerase, 2 μl of PCR buffer (10×), 0.5 μl of the forward primers, 0.5 μl of the reverse primers, 1 μl of SYBR Green dye (20×) and 1 μl of the reverse transcription products. The PCR conditions were 40 cycles at 95 °C for 10 s, 60 °C for 30 s, and 70 °C for 30 s, and melting curves were generated after the last cycle. According to previous reports^57^,^58^ and the transcriptomic information, elongation factor 1A (EF1A) was eventually chosen as the reference gene. The RT-PCR primers were designed using Primer Express software (Applied Biosystems) and are shown in Supporting Information Table S1. The threshold cycle (CT) values for each of the selected genes and EF1A were used to acquire equivalent amounts of RNA.

## Additional Information

**How to cite this article**: Xu, T. *et al*. The developmental effects of pentachlorophenol on zebrafish embryos during segmentation: A systematic view. *Sci. Rep.* **6**, 25929; doi: 10.1038/srep25929 (2016).

## Supplementary Material

Supplementary Information

## Figures and Tables

**Table 1:** Top 10 upregulated annotated mRNA transcripts in the different PCP-treated groups.

Group	Symbol	Accession	Description	BLAST	FC
10H					
1	*egln3*	NM_213310	egl-9 family hypoxia-inducible factor 3	EGLN3	97.12
2	*gapdhs*	NM_213094	glyceraldehyde-3-phosphate dehydrogenase, spermatogenic	GAPDHS	76.23
3	*eno1a*	NM_212722	enolase 1a, (alpha)	ENO1	64.19
4	*egln2*	XP_688016	egl-9 family hypoxia-inducible factor 2	EGLN2	42.74
5	*slc2a3b*	XP_002667169	solute carrier family 2 (facilitated glucose transporter), member 3b	SLC2A1	42.08
6	*igfbp1a*	NM_173283	insulin-like growth factor binding protein 1a	IGFBP1	37.75
7	*LOC100150452*	XP_001922963	sodium- and chloride-dependent creatine transporter 1-like	SLC6A8	37.07
8	*LOC799595*	XP_009292645	immunoglobulin superfamily DCC subclass member 3-like	IGDCC3	29.05
9	*LOC793364*	XP_001332264	FYVE, RhoGEF and PH domain-containing protein 4-like	FGD4	21.04
10	*slc16a9a*	NM_200410	solute carrier family 16 (monocarboxylic acid transporters), member 9a	SLC16A9	20.17
10L					
1	*LOC555573*	XP_683216	rho GTPase-activating protein 22-like	ARHGAP22	33.72
2	*rdh1*	NM_198069	retinol dehydrogenase 1	RDH5	18.83
3	*bcl2a*	NM_001030253	B-cell CLL/lymphoma 2a	BCL2	14.33
	*slc8a1a*	NM_001037102	solute carrier family 8 (sodium/calcium exchanger), member 1a	SLC8A1	14.33
5	*lenep*	NM_001302776	lens epithelial protein		13.49
	*nfatc2*	XP_003201273	nuclear factor of activated T-cells, cytoplasmic, calcineurin-dependent	NFATC2	13.49
	*cilp*	NM_001199362	cartilage intermediate layer protein, nucleotide pyrophosphohydrolase	CILP	13.49
8	*clstn2*	NM_001159839	calsyntenin 2	CLSTN2	12.65
9	*ugt5c2*	NM_001045386	UDP glucuronosyltransferase 5 family, polypeptide C2	UGT2A1	12.22
10	*cdh15*	NM_212606	cadherin 15, M-cadherin (myotubule)	CDH15	11.80
24H					
1	*mespbb*	NM_001265607	mesoderm posterior bb		218.33
2	*LOC100535991*	XP_003201455	mesoderm posterior protein 2-like	MESP2	208.25
3	*her1*	NM_131078	hairy-related 1	HES7	137.44
4	*anpepb*	NM_001089325	alanyl (membrane) aminopeptidase b	ANPEP	128.30
5	*mespaa*	NM_131551	mesoderm posterior aa	MESP2	99.29
6	*slc7a8b*	XP_001346314	solute carrier family 7 (amino acid transporter light chain, L system)	SLC7A8	94.66
7	*tbx16*	NM_131058	T-box 16	TBX6	85.61
8	*msgn1*	NM_182882	mesogenin 1	MSGN1	80.36
9	*six7*	NM_131354	SIX homeobox 7	SIX3	75.10
10	*tbx6*	NM_153666	T-box 6	TBX6	64.34
24L					
1	*slc22a5*	XP_009294016	solute carrier family 22 (organic cation/carnitine transporter), member	SLC22A5	31.81
2	*rgs9bp*	XP_691153	regulator of G-protein signaling 9 binding protein	RGS9BP	22.17
3	*her1*	NM_131078	hairy-related 1	HES7	17.74
4	*slc7a8b*	XP_001346314	solute carrier family 7 (amino acid transporter light chain, L system)	SLC7A8	17.35
5	*LOC100334535*	XP_005164589	ubl carboxyl-terminal hydrolase 18-like	USP18	14.46
6	*ftr07*	XP_698750	finTRIM family, member 7	TRIM25	13.50
7	*LOC100535991*	XP_003201455	mesoderm posterior protein 2-like	MESP2	11.57
	*mespbb*	NM_001265607	mesoderm posterior bb		11.57
8	*LOC100333218*	XP_005173582	interferon-inducible GTPase 5-like	IRGC	10.60
	*lrit3b*	NM_001145630	leucine-rich repeat, immunoglobulin-like and transmembrane domains	LRIT3	10.60
10	*vent*	NM_131700	ventral expressed homeobox	VENTX	10.51

**Table 2:** Comparison of the changes in gene expression between the RNA sequencing and qRT-PCR data (200 μg/l PCP).

	log_2_FC (10 hpf)		log_2_FC (24 hpf)	
	Sequencing	qRT-PCR	Sequencing	qRT-PCR
gapdhs	6.25	4.79	1.74	0.69
egln3	6.60	4.97	3.27	2.14
mespa	1.43	1.66	6.63	3.77
cox7a2	n/a^a^	−0.22	−1.99	−2.43
abcb11a	−1.25	−1.14	4.77	2.45
atp5ia	n/a	−0.46	−2.44	−2.81
cryba2b	n/a	0.22	−5.40	−4.52
gnb5b	1.70	1.60	−3.55	−3.97
ldha	3.25	2.00	n/a	−0.74
scn1ba	n/a	−0.24	−1.53	−2.72
her1	n/a	0.81	7.10	4.02
abcc8	1.59	1.57	−3.12	−3.67
kcnc3b	3.70	2.35	−1.90	−2.01

^a^Failed to pass the significance test when the RNA sequencing data were analyzed.

**Table 3:** Validation of the PCP concentrations before and after exposure.

Nominal	Beginning		End	
	Mean	SD	Mean	SD
200	178.00	3.26	112.57	2.95

## References

[b1] Feil R. & Fraga M. F. Epigenetics and the environment: emerging patterns and implications. Nat. Rev. Genet. 13, 97–109 (2011). 22215131 10.1038/nrg3142

[b2] Zheng W., Yu H., Wang X. & Qu W. Systematic review of pentachlorophenol occurrence in the environment and in humans in China: not a negligible health risk due to the re-emergence of schistosomiasis. Environ. Int. 42, 105–116 (2012). 21601283 10.1016/j.envint.2011.04.014

[b3] Fernández Freire P., Labrador V., Pérez Martín J. M. & Hazen M. J. Cytotoxic effects in mammalian Vero cells exposed to pentachlorophenol. Toxicology 210, 37–44 (2005). 15804456 10.1016/j.tox.2005.01.009

[b4] Naito S. . Role of active oxygen species in DNA damage by pentachlorophenol metabolites. Mutat. Res. 310, 79–88 (1994). 7523887 10.1016/0027-5107(94)90011-6

[b5] Xu T. . Pentachlorophenol exposure causes Warburg-like effects in zebrafish embryos at gastrulation stage. Toxicol. Appl. Pharmacol. 277, 183–191 (2014). 24642059 10.1016/j.taap.2014.03.004

[b6] Kimmel C. B., Ballard W. W., Kimmel S. R., Ullmann B. & Schilling T. F. Stages of embryonic development of the zebrafish. Dev. Dyn. 203, 253–310 (1995). 8589427 10.1002/aja.1002030302

[b7] Scholz S. Zebrafish embryos as an alternative model for screening of drug-induced organ toxicity. Arch. Toxicol. 87, 767–769 (2013). 23543011 10.1007/s00204-013-1044-2

[b8] Witzany G. & Baluška F. Life’s code script does not code itself. EMBO Rep. 13, 1054–1056 (2012). 23146891 10.1038/embor.2012.166 PMC3512409

[b9] Vesterlund L., Jiao H., Unneberg P., Hovatta O. & Kere J. The zebrafish transcriptome during early development. BMC Dev. Biol. 11, 30 (2011). 21609443 10.1186/1471-213X-11-30 PMC3118190

[b10] Breitholtz M. & Wollenberger L. Effects of three PBDEs on development, reproduction and population growth rate of the harpacticoid copepod Nitocra spinipes. Aquat. Toxicol. 64, 85–96 (2003). 12763669 10.1016/s0166-445x(03)00025-0

[b11] Zheng J. Energy metabolism of cancer: glycolysis versus oxidative phosphorylation [Review]. Oncol. Lett. 4, 1151–1157 (2012). 23226794 10.3892/ol.2012.928 PMC3506713

[b12] Lum J. J. . The transcription factor HIF-1alpha plays a critical role in the growth factor-dependent regulation of both aerobic and anaerobic glycolysis. Genes Dev. 21, 1037–1049 (2007). 17437992 10.1101/gad.1529107 PMC1855230

[b13] Kajimura S., Aida K. & Duan C. Insulin-like growth factor-binding protein-1 (IGFBP-1) mediates hypoxia-induced embryonic growth and developmental retardation. Proc. Natl Acad. Sci. USA 102, 1240–1245 (2005). 15644436 10.1073/pnas.0407443102 PMC545835

[b14] Henze A. T. . Prolyl hydroxylases 2 and 3 act in gliomas as protective negative feedback regulators of hypoxia-inducible factors. Cancer Res. 70, 357–366 (2010). 20028863 10.1158/0008-5472.CAN-09-1876

[b15] Balercia G. . Coenzyme Q10 treatment in infertile men with idiopathic asthenozoospermia: a placebo-controlled, double-blind randomized trial. Fertil. Steril. 91, 1785–1792 (2009). 18395716 10.1016/j.fertnstert.2008.02.119

[b16] Chai W. . Plasma coenzyme Q10 Levels and postmenopausal breast cancer risk: the Multiethnic Cohort Study. Cancer Epidemiol. Biomarkers Prev. 19, 2351–2356 (2010). 20668119 10.1158/1055-9965.EPI-10-0396 PMC3013233

[b17] Mortensen S. A. & Mortensen A. L. The mitochondria in heart failure: A target for coenzyme Q10 therapy? Clin. Pharmacol. Ther. 96, 645–647 (2014). 25399710 10.1038/clpt.2014.175

[b18] Solaini G., Baracca A., Lenaz G. & Sgarbi G. Hypoxia and mitochondrial oxidative metabolism. Biochim. Biophys. Acta 1797, 1171–1177 (2010). 20153717 10.1016/j.bbabio.2010.02.011

[b19] Liu Z. J. . Nutrient deprivation-related OXPHOS/glycolysis interconversion via HIF-1α/C-MYC pathway in U251 cells. Tumor Biol. (2015) [Epub ahead of print], doi: 10.1007/s13277-015-4479-7. 26646563

[b20] Duester G. Retinoic acid synthesis and signaling during early organogenesis. Cell 134, 921–931 (2008). 18805086 10.1016/j.cell.2008.09.002 PMC2632951

[b21] Saari J. C. Vitamin A metabolism in Rod and cone visual cycles. Annu. Rev. Nutr. 32, 125–145 (2012). 22809103 10.1146/annurev-nutr-071811-150748

[b22] Hediger M. A., Clémençon B., Burrier R. E. & Bruford E. A. The ABCs of membrane transporters in health and disease (SLC series): introduction. Mol. Aspects Med. 34, 95–107 (2013). 23506860 10.1016/j.mam.2012.12.009 PMC3853582

[b23] Gorbatenko A., Olesen C. W., Boedtkjer E. & Pedersen S. F. Regulation and roles of bicarbonate transporters in cancer. Front. Physiol. 5, 130 (2014). 24795638 10.3389/fphys.2014.00130 PMC3997025

[b24] Suhre K. . Human metabolic individuality in biomedical and pharmaceutical research. Nature 477, 54–U60 (2011). 21886157 10.1038/nature10354 PMC3832838

[b25] Hahn M. K. & Blakely R. D. The functional impact of SLC6 transporter genetic variation. Annu. Rev. Pharmacol. Toxicol. 47, 401–441 (2007). 17067279 10.1146/annurev.pharmtox.47.120505.105242

[b26] Owen L. & Sunram-Lea S. I. Metabolic agents that enhance ATP can improve cognitive functioning: a review of the evidence for glucose, oxygen, pyruvate, Creatine, and L-carnitine. Nutrients 3, 735–755 (2011). 22254121 10.3390/nu3080735 PMC3257700

[b27] Busque S. M. & Wagner C. A. Potassium restriction, high protein intake, and metabolic acidosis increase expression of the glutamine transporter SNAT3 (Slc38a3) in mouse kidney. Am. J. Physiol. Renal Physiol. 297, F440–F450 (2009). 19458124 10.1152/ajprenal.90318.2008

[b28] Kunji E. R. & Robinson A. J. The conserved substrate binding site of mitochondrial carriers. Biochim. Biophys. Acta 1757, 1237–1248 (2006). 16759636 10.1016/j.bbabio.2006.03.021

[b29] Rutenberg J., Cheng S. M. & Levin M. Early embryonic expression of ion channels and pumps in chick and Xenopus development. Dev. Dyn. 225, 469–484 (2002). 12454924 10.1002/dvdy.10180

[b30] Novak A. E. . Embryonic and larval expression of zebrafish voltage-gated sodium channel alpha-subunit genes. Dev. Dyn. 235, 1962–1973 (2006). 16615064 10.1002/dvdy.20811

[b31] Cheng S. M. & Chen I. & Levin M. K(ATP) channel activity is required for hatching in Xenopus embryos. Dev. Dyn. 225, 588–591. (2002). 12454935 10.1002/dvdy.10183

[b32] Mansergh F. . Mutation of the calcium channel gene Cacna1f disrupts calcium signaling, synaptic transmission and cellular organization in mouse retina. Hum. Mol. Genet. 14, 3035–3046 (2005). 16155113 10.1093/hmg/ddi336

[b33] An J. . Cacna1f gene decreased contractility of skeletal muscle in rat model with congenital stationary night blindness. Gene 562, 210–219 (2015). 25748727 10.1016/j.gene.2015.02.073

[b34] Reyes R. . Cloning and expression of a novel pH-sensitive two pore domain K^+^ channel from human kidney. J. Biol. Chem. 273, 30863–30869 (1998). 9812978 10.1074/jbc.273.47.30863

[b35] Leong I. U., Skinner J. R., Shelling A. N. & Love D. R. Identification and expression analysis of kcnh2 genes in the zebrafish. Biochem. Biophys. Res. Commun. 396, 817–824 (2010). 20438705 10.1016/j.bbrc.2010.04.157

[b36] Wiemuth D., Assmann M. & Gründer S. The bile acid-sensitive ion channel (BASIC), the ignored cousin of ASICs and EnaC. Channels (Austin) 8, 29–34 (2014). 24365967 10.4161/chan.27493 PMC4048340

[b37] Lin W. H., Wu C. H., Chen Y. C. & Chow W. Y. Embryonic expression of zebrafish AMPA receptor genes: zygotic gria2 alpha expression initiates at the midblastula transition. Brain Res. 1110, 46–54 (2006). 16887104 10.1016/j.brainres.2006.06.054

[b38] Fischer S. . Abcb4 acts as multixenobiotic transporter and active barrier against chemical uptake in zebrafish (Danio rerio) embryos. BMC Biol. 11, 69 (2013). 23773777 10.1186/1741-7007-11-69 PMC3765700

[b39] Lecureur V. . Cloning and expression of murine sister of P-glycoprotein reveals a more discriminating transporter than MDR1/P-glycoprotein. Mol. Pharmacol. 57, 24–35 (2000). 10617675

[b40] de Cerio O. D., Bilbao E., Cajaraville M. P. & Cancio I. Regulation of xenobiotic transporter genes in liver and brain of juvenile thicklip grey mullets (Chelon labrosus) after exposure to prestige-like fuel oil and to perfluorooctane sulfonate. Gene 498, 50–58 (2012). 22343007 10.1016/j.gene.2012.01.067

[b41] Ichida K. . Decreased extra-renal urate excretion is a common cause of hyperuricemia. Nat. Commun. 3, 764 (2012). 22473008 10.1038/ncomms1756 PMC3337984

[b42] Zaja R., Munić V. & Smital T. Cloning and mRNA expression analysis of an ABCG2 (BCRP) efflux transporter in rainbow trout (Oncorhynchus mykiss) liver and primary hepatocytes. Mar. Environ. Res. 66, 77–79 (2008). 18381223 10.1016/j.marenvres.2008.02.028

[b43] Sawada A. . Zebrafish Mesp family genes, mesp-a and mesp-b are segmentally expressed in the presomitic mesoderm, and Mesp-b confers the anterior identity to the developing somites. Development 127, 1691–1702 (2000). 10725245 10.1242/dev.127.8.1691

[b44] Gomez C. . Control of segment number in vertebrate embryos. Nature 454, 335–339 (2008). 18563087 10.1038/nature07020

[b45] Oates A. C., Morelli L. G. & Ares S. Patterning embryos with oscillations: structure, function and dynamics of the vertebrate segmentation clock. Development 139, 625–639 (2012). 22274695 10.1242/dev.063735

[b46] Wages P., Horwitz J., Ding L., Corbin R. W. & Posner M. Changes in zebrafish (Danio rerio) lens crystallin content during development. Mol. Vis. 19, 408–417 (2013). 23441112 PMC3580975

[b47] Easter S. S. & Nicola G. N. The development of vision in the zebrafish (Danio rerio). Dev. Biol. 180, 646–663 (1996). 8954734 10.1006/dbio.1996.0335

[b48] Posner M. . A proteome map of the zebrafish (Danio rerio) lens reveals similarities between zebrafish and mammalian crystallin expression. Mol. Vis. 14, 806–814 (2008). 18449354 PMC2358921

[b49] Xu T., Zhao J., Yin D., Zhao Q. & Dong B. High-throughput RNA sequencing reveals the effects of 2,2′,4,4′-tetrabromodiphenyl ether on retina and bone development of zebrafish larvae. BMC Genomics 16, 23 (2015). 25614096 10.1186/s12864-014-1194-5 PMC4312473

[b50] Hagenaars A. . Toxicity evaluation of perfluorooctane sulfonate (PFOS) in the liver of common carp (Cyprinus carpio). Aquat. Toxicol. 88, 155–163 (2008). 18501439 10.1016/j.aquatox.2008.04.002

[b51] Mazzio E. & Soliman K. F. Whole genome expression profile in neuroblastoma cells exposed to 1-methyl-4-phenylpyridine. Neurotoxicology 33, 1156–1169 (2012). 22776087 10.1016/j.neuro.2012.06.009 PMC3470775

[b52] Pujolar J. M. . Detecting genome-wide gene transcription profiles associated with high pollution burden in the critically endangered European eel. Aquat. Toxicol. 132–133, 157–164 (2013). 10.1016/j.aquatox.2013.02.012 23518471

[b53] Brown P. & Dale N. Modulation of K(+) currents in Xenopus spinal neurons by p2y receptors: a role for ATP and ADP in motor pattern generation. J. Physiol. (Lond.) 540, 843–850 (2002). 11986373 10.1113/jphysiol.2001.013192 PMC2290272

[b54] Tini M., Otulakowski G., Breitman M. L., Tsui L. C. & Giguère V. An everted repeat mediates retinoic acid induction of the gamma F-crystallin gene: evidence of a direct role for retinoids in lens development. Genes Dev. 7, 295–307 (1993). 8436299 10.1101/gad.7.2.295

[b55] Králová J., Czerny T., Spanielová H., Ratajová V. & Kozmik Z. Complex regulatory element within the gammaE- and gammaF-crystallin enhancers mediates Pax6 regulation and is required for induction by retinoic acid. Gene 286, 271–282 (2002). 11943482 10.1016/s0378-1119(02)00425-0

[b56] Mammoto T. & Ingber D. E. Mechanical control of tissue and organ development. Development 137, 1407–1420 (2010). 20388652 10.1242/dev.024166 PMC2853843

[b57] Tang R., Dodd A., Lai D., McNabb W. C. & Love D. R. (2007) Validation of zebrafish (Danio rerio) reference genes for quantitative real-time RT-PCR normalization. Acta Biochim. Biophys. Sin. (Shanghai) 39, 384–390 (2007). 17492136 10.1111/j.1745-7270.2007.00283.x PMC7110012

[b58] Curtis K. M. . EF1α and RPL13a represent normalization genes suitable for RT-qPCR analysis of bone marrow derived mesenchymal stem cells. BMC Mol. Biol. 11, 61 (2010). 20716364 10.1186/1471-2199-11-61 PMC2931506

