# Corrigendum: The developmental effects of pentachlorophenol on zebrafish embryos during segmentation: A systematic view

**DOI:** 10.1038/srep39438

**Published:** 2016-12-23

**Authors:** Ting Xu, Jing Zhao, Zhifa Xu, Ruijie Pan, Daqiang Yin

Scientific Reports 6: Article number: 2592910.1038/srep25929; published online: 05 16 2016; updated: 12 23 2016

In Supplementary Information published with this Article, the authors omitted Table S10, which contains information pertaining to the primers used in qRT-PCR. The correct Table S10 appears below.

## Figures and Tables

**Table 1:** 

Genes	Accession	Primer Sequences	Products
ef1a	NM_131263	forward: 5′-GGTGTCCTCAAGCCTGGTATG-3′	296 bp
		reverse: 5′-ATGTGAGCAGTGTGGCAATCC-3′	
gapdhs	NM_213094	forward: 5′-CAGTCGCTGATGTGTCCGTTG-3′	211 bp
		reverse: 5′-TTGTCATTGAGGGAGATGCCA-3′	
cox7a2	NM_001032728	forward: 5′-GGAGAACAAAGTCCCTAACAAGC-3′	137 bp
		reverse: 5′-CCACATAACCACTGCCAAAGATA-3′	
ldha	NM_131246	forward: 5′-GGGTTACACTTCTTGGGCTATT-3′	214 bp
		reverse: 5′-CAACTGTTTTTCCTCTTCTGGTT-3′	
egln3	NM_213310	forward: 5′-GCCGTCGTATGCTACAAGGT-3′	225 bp
		reverse: 5′-AAATAAAGTGAGGGGGAGAGGT-3′	
mespa	NM_131551	forward: 5′-GGTGAAGATGTGGAGATTTGTGA-3′	160 bp
		reverse: 5′-CACCTTGAACTGGATTCTGAGTC-3′	
her1	NM_131078	forward: 5′-TCCTTGAGAGCAGAGGCGG-3′	259 bp
		reverse: 5′-AGAATGAGCCAATCAACTACTGC-3′	
abcb11a	XM_009304974	forward: 5′-ATGGATCTCTCAACCCTATGTATG-3′	159 bp
		reverse: 5′-AGCCCTGTAAGAACTGTGAAAAG-3′	
atp5ia	NM_001172637	forward: 5′-GCTGAAATCAAAGGCGAGAG-3′	154 bp
		reverse: 5′-AATCAACCCAATGAGCAAGG-3′	
cryba2b	NM_001002584	forward: 5′-GTGGGGTACGAGTATCCTGAGT-3′	162 bp
		reverse: 5′-GGTTATCTTACTGTCGCTGTGGT-3′	
kcnc3b	NM_001195241	forward: 5′-GGATGACCCAACGGCGAG-3′	182 bp
		reverse: 5′-CAGGAACGGGCATAGCGA-3′	
gnb5b	NM_200746	forward: 5′-AATGCTCCGTCTATCCTCTTTC-3′	236 bp
		reverse: 5′-AGAGGGTGCGAGGTCAAGA-3′	
scn1ba	NM_001077539	forward: 5′-TGCTTGGCTCTGCTATGGA-3′	297 bp
		reverse: 5′-AAGGTGACGTTGAGGATGTAAAT-3′	
abcc8	NM_001172647	forward: 5′-GCCAGAGACAACGGATTAGTG-3′	242 bp
		reverse: 5′-TGTTCCCTCTGTCTGTATGCTG-3′	

