# Supplementary material for: The developmental effects of pentachlorophenol on zebrafish embryos during segmentation: A systematic view
Source: Sci Rep. 2016 May 16;6:25929. doi: 10.1038/srep25929 (PMC4867433; doi:10.1038/srep25929)
Supplement: Supplementary Information [file srep25929-s1.doc]

**The developmental effects of pentachlorophenol on zebrafish embryos during segmentation: A systematic view**

Ting Xu^1,2^, Jing Zhao^3^, Zhifa Xu^2^, Ruijie Pan^2^ & Daqiang Yin^2^

^1^Post-doctoral Research Station of Civil Engineering, Tongji University, Shanghai 200092, China

^2^Key Laboratory of Yangtze River Water Environment, Ministry of Education, College of Environmental Science and Technology, Tongji University, Shanghai 200092, China

^3^Shanghai Collaborative Innovation Centre for WEEE Recycling, WEEE Research Centre of Shanghai Second Polytechnic University, Shanghai 201209, China

Correspondence and requests for materials should be addressed to D.Y. (email: yindq@tongji.edu.cn)

Figure S1 Chromosomal distribution of the detected transcripts in all six groups. The abbreviation “MT” in X axis indicated mitochondrial chromosome.

Table S1 The most significantly changed GO terms in 10H group.

| **GOID** | **GOTerm** | **p-Value** | **Enrichment** |
| --- | --- | --- | --- |
| *BP - downregulation* | |  |  |
| GO:0007275 | multicellular organismal development | 7.1654E-16 | 2.1424425 |
| GO:0001658 | branching involved in ureteric bud morphogenesis | 6.68314E-10 | 6.4230254 |
| GO:0009952 | anterior/posterior pattern specification | 1.13368E-09 | 3.7869861 |
| GO:0048839 | inner ear development | 6.19782E-09 | 4.5878753 |
| GO:0043010 | camera-type eye development | 6.30162E-09 | 4.1185812 |
| GO:0045944 | positive regulation of transcription from RNA polymerase II promoter | 1.04022E-08 | 1.9670515 |
| GO:0030154 | cell differentiation | 1.57129E-08 | 2.0344777 |
| GO:0030198 | extracellular matrix organization | 3.92325E-08 | 2.5133578 |
| GO:0042472 | inner ear morphogenesis | 4.36321E-08 | 3.8254784 |
| GO:0030182 | neuron differentiation | 6.88601E-08 | 3.1346823 |
| GO:0001764 | neuron migration | 1.39879E-07 | 3.2288722 |
| GO:0008284 | positive regulation of cell proliferation | 5.28697E-07 | 2.0996182 |
| GO:0001656 | metanephros development | 5.94705E-07 | 4.9632469 |
| GO:0060008 | Sertoli cell differentiation | 7.49799E-07 | 10.141619 |
| GO:0030878 | thyroid gland development | 2.23396E-06 | 5.9642379 |
| GO:0030900 | forebrain development | 3.54013E-06 | 3.2115127 |
| GO:0009749 | response to glucose | 6.02452E-06 | 3.6981056 |
| GO:0045165 | cell fate commitment | 6.02452E-06 | 3.6981056 |
| GO:0045893 | positive regulation of transcription, DNA-templated | 8.19173E-06 | 1.7723915 |
| GO:0007399 | nervous system development | 8.40313E-06 | 1.8575944 |
| *BP - upregulation* | |  |  |
| GO:0055085 | transmembrane transport | 1.21607E-06 | 1.940184 |
| GO:0006096 | glycolytic process | 3.98826E-06 | 5.4782513 |
| GO:0006006 | glucose metabolic process | 1.31638E-05 | 3.0902956 |
| GO:0006811 | ion transport | 2.08303E-05 | 1.852366 |
| GO:0045944 | positive regulation of transcription from RNA polymerase II promoter | 5.05788E-05 | 1.7264393 |
| GO:0007275 | multicellular organismal development | 8.13262E-05 | 1.5605776 |
| GO:0060070 | canonical Wnt signaling pathway | 0.00021847 | 2.9167173 |
| GO:0048382 | mesendoderm development | 0.00031226 | 10.918193 |
| GO:0006816 | calcium ion transport | 0.000327941 | 2.4848302 |
| GO:0060766 | negative regulation of androgen receptor signaling pathway | 0.000381493 | 7.5852711 |
| GO:0006814 | sodium ion transport | 0.000392184 | 2.7590569 |
| GO:0030335 | positive regulation of cell migration | 0.000486776 | 2.5354471 |
| GO:0010470 | regulation of gastrulation | 0.000678598 | 13.725729 |
| GO:0001666 | response to hypoxia | 0.000755695 | 2.2187172 |
| GO:0000187 | activation of MAPK activity | 0.000855801 | 2.7503845 |
| GO:0060136 | embryonic process involved in female pregnancy | 0.000968745 | 8.006675 |
| GO:0030890 | positive regulation of B cell proliferation | 0.001043525 | 4.3672773 |
| GO:0070561 | vitamin D receptor signaling pathway | 0.001173633 | 24.020025 |
| GO:0060391 | positive regulation of SMAD protein import into nucleus | 0.001229516 | 7.5062578 |
| GO:0001707 | mesoderm formation | 0.00126308 | 3.7926355 |
| *MF - downregulation* | |  |  |
| GO:0043565 | sequence-specific DNA binding | 9.59925E-18 | 2.7645606 |
| GO:0003700 | sequence-specific DNA binding transcription factor activity | 2.71402E-14 | 2.2101639 |
| GO:0008083 | growth factor activity | 1.77545E-07 | 3.2616538 |
| GO:0005509 | calcium ion binding | 1.95036E-05 | 1.7111865 |
| GO:0005102 | receptor binding | 2.17894E-05 | 2.0864105 |
| GO:0000976 | transcription regulatory region sequence-specific DNA binding | 2.18334E-05 | 4.0435571 |
| GO:0019841 | retinol binding | 3.98898E-05 | 7.8714579 |
| GO:0016918 | retinal binding | 6.88269E-05 | 7.1558708 |
| GO:0005178 | integrin binding | 8.67386E-05 | 2.9674147 |
| GO:0071837 | HMG box domain binding | 0.000103783 | 5.7131549 |
| GO:0008201 | heparin binding | 0.000104125 | 2.6012002 |
| GO:0005501 | retinoid binding | 0.000105363 | 8.1029714 |
| GO:0046983 | protein dimerization activity | 0.000122416 | 2.3614374 |
| GO:0070888 | E-box binding | 0.00012723 | 5.5346188 |
| GO:0003677 | DNA binding | 0.000132786 | 1.3774277 |
| GO:0008289 | lipid binding | 0.000352875 | 2.0679254 |
| GO:0001046 | core promoter sequence-specific DNA binding | 0.000383406 | 6.261387 |
| GO:0044212 | transcription regulatory region DNA binding | 0.000493194 | 2.1424331 |
| GO:0030984 | kininogen binding | 0.001348459 | 7.5687095 |
| GO:0017127 | cholesterol transporter activity | 0.001521111 | 4.7500177 |
| *MF - upregulation* | |  |  |
| GO:0005216 | ion channel activity | 1.45909E-05 | 2.6977761 |
| GO:0005506 | iron ion binding | 0.000211572 | 2.4526867 |
| GO:0004971 | alpha-amino-3-hydroxy-5-methyl-4-isoxazole propionate selective glutamate receptor activity | 0.000703591 | 13.588798 |
| GO:0004714 | transmembrane receptor protein tyrosine kinase activity | 0.000721623 | 3.4877916 |
| GO:0005215 | transporter activity | 0.000827445 | 1.9989319 |
| GO:0051213 | dioxygenase activity | 0.000903331 | 3.1707196 |
| GO:0004872 | receptor activity | 0.000957723 | 1.9298228 |
| GO:0004383 | guanylate cyclase activity | 0.001011341 | 7.926799 |
| GO:0003707 | steroid hormone receptor activity | 0.001057869 | 3.3111945 |
| GO:0031687 | A2A adenosine receptor binding | 0.001206854 | 23.780397 |
| GO:0042803 | protein homodimerization activity | 0.001904128 | 1.6024526 |
| GO:0070644 | vitamin D response element binding | 0.001935272 | 9.5121588 |
| GO:0043125 | ErbB-3 class receptor binding | 0.002048182 | 17.835298 |
| GO:0016705 | oxidoreductase activity, acting on paired donors, with incorporation or reduction of molecular oxygen | 0.002518372 | 2.6422663 |
| GO:0016706 | oxidoreductase activity, acting on paired donors, with incorporation or reduction of molecular oxygen, 2-oxoglutarate as one donor, and incorporation of one atom each of oxygen into both donors | 0.002705286 | 4.1615695 |
| GO:0046332 | SMAD binding | 0.002830877 | 3.6585226 |
| GO:0003700 | sequence-specific DNA binding transcription factor activity | 0.00302651 | 1.4442338 |
| GO:0004713 | protein tyrosine kinase activity | 0.003093361 | 2.3059779 |
| GO:0005244 | voltage-gated ion channel activity | 0.003542475 | 2.1509907 |
| GO:0008331 | high voltage-gated calcium channel activity | 0.004174327 | 7.3170452 |
| *CC- downregulation* | |  |  |
| GO:0005576 | extracellular region | 2.69288E-18 | 2.0880871 |
| GO:0005615 | extracellular space | 1.04008E-14 | 2.4273429 |
| GO:0005578 | proteinaceous extracellular matrix | 8.46176E-08 | 2.4197404 |
| GO:0031012 | extracellular matrix | 2.36767E-07 | 2.5682786 |
| GO:0005667 | transcription factor complex | 2.79344E-06 | 2.4255017 |
| GO:0031093 | platelet alpha granule lumen | 3.60118E-05 | 4.0935562 |
| GO:0005788 | endoplasmic reticulum lumen | 6.99851E-05 | 2.405862 |
| GO:0009986 | cell surface | 0.000140885 | 1.8774316 |
| GO:0005902 | microvillus | 0.000147284 | 3.74526 |
| GO:0042627 | chylomicron | 0.000311404 | 6.5301969 |
| GO:0034361 | very-low-density lipoprotein particle | 0.000607946 | 5.7139223 |
| GO:0005604 | basement membrane | 0.000953694 | 2.634029 |
| GO:0030863 | cortical cytoskeleton | 0.001841349 | 4.5711378 |
| GO:0030017 | sarcomere | 0.002005586 | 2.7384697 |
| GO:0005581 | collagen trimer | 0.002554845 | 2.2968279 |
| GO:0035631 | CD40 receptor complex | 0.00293436 | 4.1555798 |
| GO:0005887 | integral component of plasma membrane | 0.002952311 | 1.3806425 |
| GO:0031983 | vesicle lumen | 0.003787544 | 7.8362362 |
| GO:0034359 | mature chylomicron | 0.003787544 | 7.8362362 |
| GO:0034360 | chylomicron remnant | 0.003787544 | 7.8362362 |
| *CC- upregulation* | |  |  |
| GO:0005886 | plasma membrane | 0.000239885 | 1.3046643 |
| GO:0016323 | basolateral plasma membrane | 0.002114789 | 2.1599161 |
| GO:0009897 | external side of plasma membrane | 0.002954579 | 2.1390301 |
| GO:0045202 | synapse | 0.002974037 | 1.6764303 |
| GO:0030054 | cell junction | 0.003284102 | 1.5046633 |
| GO:0043197 | dendritic spine | 0.003612817 | 2.5225362 |
| GO:0008021 | synaptic vesicle | 0.005200514 | 2.4052089 |
| GO:0043235 | receptor complex | 0.007857393 | 3.3413902 |
| GO:0005887 | integral component of plasma membrane | 0.008441447 | 1.3633269 |
| GO:0097136 | Bcl-2 family protein complex | 0.008463806 | 8.9501524 |
| GO:0031225 | anchored component of membrane | 0.009522864 | 2.2162282 |
| GO:0030425 | dendrite | 0.011120301 | 1.7482251 |
| GO:0000932 | cytoplasmic mRNA processing body | 0.014412015 | 2.6892477 |
| GO:0014069 | postsynaptic density | 0.019507548 | 1.9426687 |
| GO:0030018 | Z disc | 0.021165035 | 1.9203392 |
| GO:0005642 | annulate lamellae | 0.021839447 | 11.933537 |
| GO:0032437 | cuticular plate | 0.021839447 | 11.933537 |
| GO:0045211 | postsynaptic membrane | 0.026026534 | 1.6632107 |
| GO:0034707 | chloride channel complex | 0.031296651 | 2.7019328 |
| GO:0031410 | cytoplasmic vesicle | 0.031696606 | 1.4387952 |

Table S2 The most significantly changed GO terms in 10L group.

| **GOID** | **GOTerm** | **p-Value** | **Enrichment** |
| --- | --- | --- | --- |
| *BP - downregulation* | |  |  |
| GO:0007268 | synaptic transmission | 2.608E-07 | 3.3889499 |
| GO:0006811 | ion transport | 4.811E-05 | 2.4525734 |
| GO:0055085 | transmembrane transport | 0.0001142 | 2.2858598 |
| GO:0042662 | negative regulation of mesodermal cell fate specification | 0.0002315 | 34.642599 |
| GO:0006334 | nucleosome assembly | 0.0002552 | 4.6534835 |
| GO:0030104 | water homeostasis | 0.0004451 | 25.981949 |
| GO:0019228 | neuronal action potential | 0.0004919 | 12.597309 |
| GO:0071805 | potassium ion transmembrane transport | 0.0005753 | 4.6190132 |
| GO:0051246 | regulation of protein metabolic process | 0.0007549 | 20.78556 |
| GO:0070654 | sensory epithelium regeneration | 0.0011754 | 17.3213 |
| GO:0090082 | positive regulation of heart induction by negative regulation of canonical Wnt signaling pathway | 0.0012042 | 69.285199 |
| GO:1901296 | negative regulation of canonical Wnt signaling pathway involved in cardiac muscle cell fate commitment | 0.0012042 | 69.285199 |
| GO:0007586 | digestion | 0.0013752 | 6.6620383 |
| GO:0035584 | calcium-mediated signaling using intracellular calcium source | 0.0013918 | 9.2380265 |
| GO:0060323 | head morphogenesis | 0.0017192 | 14.846828 |
| GO:0044117 | growth of symbiont in host | 0.0019877 | 46.190132 |
| GO:0032827 | negative regulation of natural killer cell differentiation involved in immune response | 0.0019877 | 46.190132 |
| GO:0002041 | intussusceptive angiogenesis | 0.0019877 | 46.190132 |
| GO:0016126 | sterol biosynthetic process | 0.0023312 | 7.9183084 |
| GO:0007602 | phototransduction | 0.0028161 | 5.587516 |
| *BP - upregulation* | |  |  |
| GO:0030198 | extracellular matrix organization | 9.189E-10 | 3.5770007 |
| GO:0007155 | cell adhesion | 5.413E-09 | 2.5279241 |
| GO:0007275 | multicellular organismal development | 1.928E-07 | 2.0036608 |
| GO:0001525 | angiogenesis | 1.34E-06 | 3.0133121 |
| GO:0007411 | axon guidance | 5.724E-06 | 2.5739265 |
| GO:0007599 | hemostasis | 5.796E-06 | 6.1935105 |
| GO:0045165 | cell fate commitment | 5.893E-06 | 5.1015417 |
| GO:0030154 | cell differentiation | 2.36E-05 | 2.0745254 |
| GO:0007596 | blood coagulation | 2.669E-05 | 2.2413578 |
| GO:0048839 | inner ear development | 4.582E-05 | 4.4400231 |
| GO:0007156 | homophilic cell adhesion via plasma membrane adhesion molecules | 4.95E-05 | 3.1642699 |
| GO:0032332 | positive regulation of chondrocyte differentiation | 7.466E-05 | 10.134835 |
| GO:0030182 | neuron differentiation | 7.956E-05 | 3.1600643 |
| GO:0042632 | cholesterol homeostasis | 9.554E-05 | 5.4633097 |
| GO:0045540 | regulation of cholesterol biosynthetic process | 0.0001155 | 12.950067 |
| GO:0048754 | branching morphogenesis of an epithelial tube | 0.0001172 | 6.0941494 |
| GO:0050885 | neuromuscular process controlling balance | 0.0001181 | 5.2977549 |
| GO:0042472 | inner ear morphogenesis | 0.0001207 | 3.7136223 |
| GO:0007229 | integrin-mediated signaling pathway | 0.0001375 | 4.2312102 |
| GO:0030517 | negative regulation of axon extension | 0.0001596 | 8.6333783 |
| *MF - downregulation* | |  |  |
| GO:0005249 | voltage-gated potassium channel activity | 7.186E-05 | 6.4060829 |
| GO:0005216 | ion channel activity | 7.988E-05 | 3.849033 |
| GO:0005215 | transporter activity | 0.0007767 | 2.8595269 |
| GO:0042802 | identical protein binding | 0.0015224 | 2.1729885 |
| GO:0008745 | N-acetylmuramoyl-L-alanine amidase activity | 0.0019238 | 46.977941 |
| GO:0016019 | peptidoglycan receptor activity | 0.0019238 | 46.977941 |
| GO:0046982 | protein heterodimerization activity | 0.0021429 | 2.3126207 |
| GO:0019838 | growth factor binding | 0.0021461 | 5.9717722 |
| GO:0005516 | calmodulin binding | 0.0025165 | 3.0637788 |
| GO:0005501 | retinoid binding | 0.0026635 | 12.435337 |
| GO:0003872 | 6-phosphofructokinase activity | 0.0028587 | 35.233456 |
| GO:0008395 | steroid hydroxylase activity | 0.0030748 | 11.744485 |
| GO:0001567 | cholesterol 25-hydroxylase activity | 0.0039646 | 28.186765 |
| GO:0005251 | delayed rectifier potassium channel activity | 0.0040286 | 6.7111345 |
| GO:0005267 | potassium channel activity | 0.0042778 | 4.1861532 |
| GO:0048019 | receptor antagonist activity | 0.0052365 | 23.488971 |
| GO:0004924 | oncostatin-M receptor activity | 0.0052365 | 23.488971 |
| GO:0004923 | leukemia inhibitory factor receptor activity | 0.0052365 | 23.488971 |
| GO:0004897 | ciliary neurotrophic factor receptor activity | 0.0066697 | 20.133403 |
| GO:0070061 | fructose binding | 0.0066697 | 20.133403 |
| *MF - upregulation* | |  |  |
| GO:0005509 | calcium ion binding | 6.609E-08 | 2.3521914 |
| GO:0043565 | sequence-specific DNA binding | 1.755E-05 | 2.1221651 |
| GO:0005216 | ion channel activity | 0.0001259 | 2.9225908 |
| GO:0000980 | RNA polymerase II distal enhancer sequence-specific DNA binding | 0.0001641 | 8.5873656 |
| GO:0005102 | receptor binding | 0.0002256 | 2.3279003 |
| GO:0005543 | phospholipid binding | 0.0002282 | 2.2834586 |
| GO:0017127 | cholesterol transporter activity | 0.0002302 | 7.9951335 |
| GO:0005109 | frizzled binding | 0.0003767 | 5.8804786 |
| GO:0004872 | receptor activity | 0.0003885 | 2.3266815 |
| GO:0003700 | sequence-specific DNA binding transcription factor activity | 0.0004402 | 1.7038991 |
| GO:0042802 | identical protein binding | 0.0005649 | 1.92932 |
| GO:0005319 | lipid transporter activity | 0.001375 | 6.9005616 |
| GO:0004971 | alpha-amino-3-hydroxy-5-methyl-4-isoxazole propionate selective glutamate receptor activity | 0.0017041 | 16.561348 |
| GO:0005201 | extracellular matrix structural constituent | 0.0020439 | 3.4434486 |
| GO:0005089 | Rho guanyl-nucleotide exchange factor activity | 0.0021744 | 3.4096893 |
| GO:0005178 | integrin binding | 0.0026223 | 3.0669163 |
| GO:0004222 | metalloendopeptidase activity | 0.00293 | 3.250358 |
| GO:0005245 | voltage-gated calcium channel activity | 0.0031948 | 4.5462524 |
| GO:0005520 | insulin-like growth factor binding | 0.0032905 | 5.5204493 |
| GO:0038085 | vascular endothelial growth factor binding | 0.0037117 | 38.643145 |
| *CC - downregulation* | |  |  |
| GO:0000786 | nucleosome | 2.121E-05 | 6.583651 |
| GO:0005887 | integral component of plasma membrane | 0.0002827 | 1.967715 |
| GO:0005886 | plasma membrane | 0.0003676 | 1.5239669 |
| GO:0005576 | extracellular region | 0.0004207 | 1.7660853 |
| GO:0008076 | voltage-gated potassium channel complex | 0.000437 | 5.5757835 |
| GO:0043005 | neuron projection | 0.0010466 | 2.8942621 |
| GO:0045211 | postsynaptic membrane | 0.0011983 | 2.9974346 |
| GO:0005945 | 6-phosphofructokinase complex | 0.002765 | 35.844322 |
| GO:0031093 | platelet alpha granule lumen | 0.0033389 | 5.3498989 |
| GO:0030018 | Z disc | 0.0042646 | 3.2960296 |
| GO:0016021 | integral component of membrane | 0.0047296 | 1.3683526 |
| GO:0043197 | dendritic spine | 0.0095915 | 3.4970071 |
| GO:0034774 | secretory granule lumen | 0.01151 | 14.337729 |
| GO:0045178 | basal part of cell | 0.0134781 | 13.034299 |
| GO:0005694 | chromosome | 0.0165396 | 2.1033343 |
| GO:0030426 | growth cone | 0.0178106 | 3.0290977 |
| GO:0044224 | juxtaparanode region of axon | 0.0178134 | 11.029022 |
| GO:0043025 | neuronal cell body | 0.0199745 | 2.2126125 |
| GO:0061202 | clathrin-sculpted gamma-aminobutyric acid transport vesicle membrane | 0.0252535 | 8.9610806 |
| GO:0016020 | membrane | 0.0256371 | 1.2390177 |
| *CC - upregulation* | |  |  |
| GO:0005576 | extracellular region | 2.777E-13 | 2.2810902 |
| GO:0031012 | extracellular matrix | 3.84E-12 | 4.3373365 |
| GO:0005578 | proteinaceous extracellular matrix | 5.934E-12 | 3.8358572 |
| GO:0009986 | cell surface | 5.087E-08 | 2.9638466 |
| GO:0005886 | plasma membrane | 4.855E-07 | 1.5596815 |
| GO:0005788 | endoplasmic reticulum lumen | 7.523E-07 | 3.7100782 |
| GO:0005615 | extracellular space | 1.197E-06 | 2.192319 |
| GO:0030054 | cell junction | 3.077E-06 | 2.1314619 |
| GO:0045211 | postsynaptic membrane | 2.205E-05 | 2.9473792 |
| GO:0045202 | synapse | 2.39E-05 | 2.3453729 |
| GO:0005604 | basement membrane | 0.0001494 | 3.877301 |
| GO:0001917 | photoreceptor inner segment | 0.0002069 | 6.5646174 |
| GO:0005581 | collagen trimer | 0.0002372 | 3.4472326 |
| GO:0001750 | photoreceptor outer segment | 0.000277 | 5.3034347 |
| GO:0009897 | external side of plasma membrane | 0.0003076 | 2.9018794 |
| GO:0031983 | vesicle lumen | 0.0003428 | 15.379961 |
| GO:0034359 | mature chylomicron | 0.0003428 | 15.379961 |
| GO:0034360 | chylomicron remnant | 0.0003428 | 15.379961 |
| GO:0005796 | Golgi lumen | 0.000376 | 4.0473581 |
| GO:0005865 | striated muscle thin filament | 0.0005983 | 12.816634 |

Table S3 The most significantly changed GO terms in 24H group.

| **GOID** | **GOTerm** | **P-Value** | **Enrichment** |
| --- | --- | --- | --- |
| *BP - downregulation* | |  |  |
| GO:0044281 | small molecule metabolic process | 5.684E-11 | 1.557694 |
| GO:0006521 | regulation of cellular amino acid metabolic process | 1.184E-09 | 4.61656884 |
| GO:0007268 | synaptic transmission | 2.957E-09 | 1.90684365 |
| GO:0034641 | cellular nitrogen compound metabolic process | 3.296E-09 | 2.5233027 |
| GO:0006977 | DNA damage response, signal transduction by p53 class mediator resulting in cell cycle arrest | 4.681E-09 | 3.9116063 |
| GO:0007601 | visual perception | 2.273E-08 | 2.08136833 |
| GO:0051436 | negative regulation of ubiquitin-protein ligase activity involved in mitotic cell cycle | 3.548E-08 | 3.81368731 |
| GO:0007399 | nervous system development | 4.501E-08 | 1.72905449 |
| GO:0002479 | antigen processing and presentation of exogenous peptide antigen via MHC class I, TAP-dependent | 1.028E-07 | 3.32711341 |
| GO:0031145 | anaphase-promoting complex-dependent proteasomal ubiquitin-dependent protein catabolic process | 1.818E-07 | 3.30220454 |
| GO:0051437 | positive regulation of ubiquitin-protein ligase activity involved in mitotic cell cycle | 1.908E-07 | 3.46242663 |
| GO:0051439 | regulation of ubiquitin-protein ligase activity involved in mitotic cell cycle | 2.091E-07 | 3.35698648 |
| GO:0042590 | antigen processing and presentation of exogenous peptide antigen via MHC class I | 4.322E-07 | 3.07934964 |
| GO:0002474 | antigen processing and presentation of peptide antigen via MHC class I | 1.632E-06 | 2.62453756 |
| GO:0042981 | regulation of apoptotic process | 3.969E-06 | 2.011505 |
| GO:0002088 | lens development in camera-type eye | 4.558E-06 | 2.75183712 |
| GO:0007269 | neurotransmitter secretion | 1.392E-05 | 2.79092571 |
| GO:0006810 | transport | 1.854E-05 | 1.30953378 |
| GO:0030182 | neuron differentiation | 1.969E-05 | 2.05647158 |
| GO:0070309 | lens fiber cell morphogenesis | 2.077E-05 | 3.75920606 |
| *BP - upregulation* | |  |  |
| GO:0007275 | multicellular organismal development | 6.894E-18 | 1.96638845 |
| GO:0045944 | positive regulation of transcription from RNA polymerase II promoter | 7.778E-14 | 2.03455015 |
| GO:0030154 | cell differentiation | 7.146E-11 | 1.95811496 |
| GO:0001756 | somitogenesis | 1.024E-10 | 3.79108723 |
| GO:0009952 | anterior/posterior pattern specification | 3.992E-10 | 3.22571122 |
| GO:0045893 | positive regulation of transcription, DNA-templated | 1.056E-09 | 1.87408463 |
| GO:0006355 | regulation of transcription, DNA-templated | 2.164E-09 | 1.47035089 |
| GO:0036342 | post-anal tail morphogenesis | 1.351E-08 | 5.9177947 |
| GO:0000122 | negative regulation of transcription from RNA polymerase II promoter | 1.614E-08 | 1.84678685 |
| GO:0031290 | retinal ganglion cell axon guidance | 5.332E-08 | 4.57416427 |
| GO:0090090 | negative regulation of canonical Wnt signaling pathway | 7.982E-08 | 3.19744411 |
| GO:0045892 | negative regulation of transcription, DNA-templated | 9.869E-08 | 1.91440151 |
| GO:0001947 | heart looping | 1.472E-07 | 3.10128038 |
| GO:0043049 | otic placode formation | 2.608E-07 | 7.38437861 |
| GO:0007498 | mesoderm development | 2.953E-07 | 4.66595352 |
| GO:0007389 | pattern specification process | 3.522E-07 | 2.96741216 |
| GO:0009953 | dorsal/ventral pattern formation | 3.604E-07 | 2.9082313 |
| GO:0008284 | positive regulation of cell proliferation | 3.779E-07 | 1.87622173 |
| GO:0060070 | canonical Wnt signaling pathway | 4.053E-07 | 2.94621636 |
| GO:0042074 | cell migration involved in gastrulation | 6.054E-07 | 3.73276281 |
| *MF - downregulation* | |  |  |
| GO:0005212 | structural constituent of eye lens | 2.718E-09 | 4.02237866 |
| GO:0005215 | transporter activity | 6.214E-07 | 1.95872352 |
| GO:0005509 | calcium ion binding | 2.022E-06 | 1.53581731 |
| GO:0004298 | threonine-type endopeptidase activity | 3.293E-06 | 5.48506181 |
| GO:0005344 | oxygen transporter activity | 4.222E-05 | 5.26565934 |
| GO:0048306 | calcium-dependent protein binding | 5.148E-05 | 2.97037194 |
| GO:0004175 | endopeptidase activity | 6.171E-05 | 3.02127995 |
| GO:0009055 | electron carrier activity | 0.0001452 | 2.3349254 |
| GO:0005179 | hormone activity | 0.0001656 | 2.3136988 |
| GO:0005504 | fatty acid binding | 0.0002122 | 4.21252747 |
| GO:0019825 | oxygen binding | 0.0002176 | 3.26552517 |
| GO:0004601 | peroxidase activity | 0.0003685 | 3.9004884 |
| GO:0016491 | oxidoreductase activity | 0.0003953 | 1.43062434 |
| GO:0005216 | ion channel activity | 0.0004927 | 1.76996953 |
| GO:0019841 | retinol binding | 0.0005526 | 4.38804945 |
| GO:0046983 | protein dimerization activity | 0.0008314 | 1.75521978 |
| GO:0020037 | heme binding | 0.000969 | 1.85551805 |
| GO:0005184 | neuropeptide hormone activity | 0.0009795 | 3.98913586 |
| GO:0051920 | peroxiredoxin activity | 0.0016712 | 6.58207418 |
| GO:0030899 | calcium-dependent ATPase activity | 0.0018214 | 8.7760989 |
| *MF - upregulation* | |  |  |
| GO:0043565 | sequence-specific DNA binding | 5.357E-16 | 2.27348815 |
| GO:0003700 | sequence-specific DNA binding transcription factor activity | 1.925E-15 | 1.98816857 |
| GO:0003705 | RNA polymerase II distal enhancer sequence-specific DNA binding transcription factor activity | 2.453E-09 | 3.45764498 |
| GO:0044212 | transcription regulatory region DNA binding | 1.237E-08 | 2.60570826 |
| GO:0016705 | oxidoreductase activity, acting on paired donors, with incorporation or reduction of molecular oxygen | 1.757E-06 | 3.02213414 |
| GO:0008083 | growth factor activity | 9.033E-06 | 2.42507697 |
| GO:0003677 | DNA binding | 2.463E-05 | 1.33290758 |
| GO:0016712 | oxidoreductase activity, acting on paired donors, with incorporation or reduction of molecular oxygen, reduced flavin or flavoprotein as one donor, and incorporation of one atom of oxygen | 2.608E-05 | 4.15661867 |
| GO:0004812 | aminoacyl-tRNA ligase activity | 2.608E-05 | 4.15661867 |
| GO:0051213 | dioxygenase activity | 9.041E-05 | 2.84497455 |
| GO:0070330 | aromatase activity | 0.0001079 | 3.79329941 |
| GO:0016706 | oxidoreductase activity, acting on paired donors, with incorporation or reduction of molecular oxygen, 2-oxoglutarate as one donor, and incorporation of one atom each of oxygen into both donors | 0.0001311 | 3.96264313 |
| GO:0003707 | steroid hormone receptor activity | 0.0001361 | 2.93243309 |
| GO:0048018 | receptor agonist activity | 0.0001533 | 5.0803117 |
| GO:0005102 | receptor binding | 0.0001668 | 1.73342684 |
| GO:0005506 | iron ion binding | 0.0001924 | 2.02301202 |
| GO:0042802 | identical protein binding | 0.0002803 | 1.53975967 |
| GO:0002161 | aminoacyl-tRNA editing activity | 0.0003238 | 7.11243639 |
| GO:0005201 | extracellular matrix structural constituent | 0.0003534 | 2.53512584 |
| GO:0046983 | protein dimerization activity | 0.000423 | 1.95083969 |
| *CC - downregulation* | |  |  |
| GO:0000502 | proteasome complex | 5.196E-09 | 3.89277793 |
| GO:0045202 | synapse | 2.682E-07 | 1.77489567 |
| GO:0008021 | synaptic vesicle | 2.457E-06 | 2.5714098 |
| GO:0005839 | proteasome core complex | 3.493E-06 | 5.45578724 |
| GO:0043025 | neuronal cell body | 1.695E-05 | 1.83206683 |
| GO:0030424 | axon | 2.567E-05 | 1.8934791 |
| GO:0022624 | proteasome accessory complex | 4.426E-05 | 5.23755575 |
| GO:0030672 | synaptic vesicle membrane | 4.573E-05 | 3.00068298 |
| GO:0005829 | cytosol | 0.0001343 | 1.22666945 |
| GO:0005576 | extracellular region | 0.0001513 | 1.28152026 |
| GO:0005833 | hemoglobin complex | 0.0003041 | 4.84958866 |
| GO:0033162 | melanosome membrane | 0.0003341 | 6.34855243 |
| GO:0005615 | extracellular space | 0.0006031 | 1.36873259 |
| GO:0030425 | dendrite | 0.0006734 | 1.6402112 |
| GO:0005578 | proteinaceous extracellular matrix | 0.001247 | 1.51362458 |
| GO:0005782 | peroxisomal matrix | 0.001299 | 2.78593391 |
| GO:0005739 | mitochondrion | 0.001546 | 1.24075654 |
| GO:0016529 | sarcoplasmic reticulum | 0.0021887 | 2.23827169 |
| GO:0005887 | integral component of plasma membrane | 0.0023951 | 1.26276381 |
| GO:0043005 | neuron projection | 0.0026104 | 1.5452416 |
| *CC - upregulation* | |  |  |
| GO:0005615 | extracellular space | 2.045E-07 | 1.70121572 |
| GO:0031012 | extracellular matrix | 5.172E-06 | 2.04851596 |
| GO:0005578 | proteinaceous extracellular matrix | 8.797E-06 | 1.88617405 |
| GO:0031090 | organelle membrane | 1.129E-05 | 2.75858806 |
| GO:0005887 | integral component of plasma membrane | 1.376E-05 | 1.46816757 |
| GO:0005576 | extracellular region | 4.996E-05 | 1.35740047 |
| GO:0016324 | apical plasma membrane | 6.977E-05 | 1.97750788 |
| GO:0005730 | nucleolus | 7.001E-05 | 1.36105333 |
| GO:0005604 | basement membrane | 0.0002039 | 2.46385296 |
| GO:0043186 | P granule | 0.0002164 | 7.7742027 |
| GO:0009986 | cell surface | 0.0002605 | 1.65433182 |
| GO:0005667 | transcription factor complex | 0.0004539 | 1.78401205 |
| GO:0033391 | chromatoid body | 0.0011273 | 6.66360232 |
| GO:0005911 | cell-cell junction | 0.0014163 | 1.97725387 |
| GO:0005634 | nucleus | 0.0016624 | 1.15861538 |
| GO:0030057 | desmosome | 0.0021944 | 4.07220141 |
| GO:0000790 | nuclear chromatin | 0.0053605 | 1.93356326 |
| GO:0016281 | eukaryotic translation initiation factor 4F complex | 0.0061949 | 4.31174267 |
| GO:0045121 | membrane raft | 0.0062273 | 1.75417907 |
| GO:0031093 | platelet alpha granule lumen | 0.0070354 | 2.3703859 |

Table S4 The most significantly changed GO terms in 24L group.

| **GOID** | **GOTerm** | **p-Value** | **Enrichment** |
| --- | --- | --- | --- |
| *BP - downregulation* | |  |  |
| GO:0002088 | lens development in camera-type eye | 8.36828E-18 | 9.95720624 |
| GO:0070309 | lens fiber cell morphogenesis | 3.7741E-14 | 15.00938478 |
| GO:0007601 | visual perception | 2.20027E-12 | 4.056208503 |
| GO:0070307 | lens fiber cell development | 2.97823E-12 | 12.15045434 |
| GO:0001654 | eye development | 3.62371E-10 | 6.734980348 |
| GO:0050806 | positive regulation of synaptic transmission | 2.40334E-05 | 13.13321168 |
| GO:0043010 | camera-type eye development | 6.46374E-05 | 3.742798239 |
| GO:0006805 | xenobiotic metabolic process | 7.93648E-05 | 3.166873738 |
| GO:0001523 | retinoid metabolic process | 9.9161E-05 | 4.080221104 |
| GO:0072049 | comma-shaped body morphogenesis | 0.000106806 | 13.4699607 |
| GO:0072050 | S-shaped body morphogenesis | 0.000106806 | 13.4699607 |
| GO:0006704 | glucocorticoid biosynthetic process | 0.000249788 | 17.51094891 |
| GO:0016125 | sterol metabolic process | 0.000273726 | 7.782643958 |
| GO:0008202 | steroid metabolic process | 0.000286539 | 3.2046181 |
| GO:0060992 | response to fungicide | 0.000352787 | 15.56528792 |
| GO:0045471 | response to ethanol | 0.000443473 | 3.416770518 |
| GO:0007268 | synaptic transmission | 0.000701536 | 1.966809478 |
| GO:0048708 | astrocyte differentiation | 0.000815601 | 7.95952223 |
| GO:0007603 | phototransduction, visible light | 0.000826789 | 2.995293892 |
| GO:0014070 | response to organic cyclic compound | 0.000826789 | 2.995293892 |
| *BP - upregulation* | |  |  |
| GO:0001756 | somitogenesis | 2.78984E-08 | 6.414438503 |
| GO:0007596 | blood coagulation | 2.2967E-05 | 2.467091732 |
| GO:0006096 | glycolytic process | 3.66272E-05 | 7.202176564 |
| GO:0023019 | signal transduction involved in regulation of gene expression | 5.14833E-05 | 10.61700166 |
| GO:0007498 | mesoderm development | 0.000140793 | 6.907856849 |
| GO:0050796 | regulation of insulin secretion | 0.000228027 | 4.761232703 |
| GO:0030917 | midbrain-hindbrain boundary development | 0.000252175 | 10.2631016 |
| GO:0036342 | post-anal tail morphogenesis | 0.000279121 | 7.50958654 |
| GO:0019369 | arachidonic acid metabolic process | 0.000456759 | 4.829694873 |
| GO:0006000 | fructose metabolic process | 0.000517848 | 12.82887701 |
| GO:0042593 | glucose homeostasis | 0.000790609 | 4.414237249 |
| GO:0006006 | glucose metabolic process | 0.000918756 | 3.300997592 |
| GO:0051497 | negative regulation of stress fiber assembly | 0.001042052 | 19.24331551 |
| GO:0030198 | extracellular matrix organization | 0.001289292 | 2.362350753 |
| GO:0001707 | mesoderm formation | 0.001356918 | 5.401632423 |
| GO:0030903 | notochord development | 0.001699928 | 6.414438503 |
| GO:0015698 | inorganic anion transport | 0.001754723 | 15.39465241 |
| GO:0001947 | heart looping | 0.001861227 | 3.472477986 |
| GO:0017144 | drug metabolic process | 0.001934862 | 8.55258467 |
| GO:0006094 | gluconeogenesis | 0.00201381 | 4.966016905 |
| *MF - downregulation* | |  |  |
| GO:0005212 | structural constituent of eye lens | 1.8626E-22 | 14.92757009 |
| GO:0005179 | hormone activity | 4.3298E-07 | 5.211079014 |
| GO:0005506 | iron ion binding | 4.9823E-05 | 3.052453795 |
| GO:0020037 | heme binding | 8.7482E-05 | 3.275535381 |
| GO:0005184 | neuropeptide hormone activity | 9.3125E-05 | 9.770773152 |
| GO:0001972 | retinoic acid binding | 0.00039895 | 7.165233645 |
| GO:0005102 | receptor binding | 0.00063555 | 2.158202905 |
| GO:0050786 | RAGE receptor binding | 0.00150219 | 17.91308411 |
| GO:0016595 | glutamate binding | 0.00188663 | 8.956542056 |
| GO:0001078 | RNA polymerase II core promoter proximal region sequence-specific DNA binding transcription factor activity involved in negative regulation of transcription | 0.00197374 | 4.323847889 |
| GO:0019825 | oxygen binding | 0.00211448 | 4.999000217 |
| GO:0015020 | glucuronosyltransferase activity | 0.00211448 | 4.999000217 |
| GO:0004497 | monooxygenase activity | 0.0021491 | 2.963066545 |
| GO:0016705 | oxidoreductase activity, acting on paired donors, with incorporation or reduction of molecular oxygen | 0.00268166 | 3.06206566 |
| GO:0046982 | protein heterodimerization activity | 0.00283416 | 1.867387147 |
| GO:0048306 | calcium-dependent protein binding | 0.00353875 | 3.858202732 |
| GO:0016888 | endodeoxyribonuclease activity, producing 5'-phosphomonoesters | 0.00369947 | 11.94205607 |
| GO:0019841 | retinol binding | 0.00379269 | 7.165233645 |
| GO:0008020 | G-protein coupled photoreceptor activity | 0.00379269 | 7.165233645 |
| GO:0005344 | oxygen transporter activity | 0.00379269 | 7.165233645 |
| *MF - upregulation* | |  |  |
| GO:0005201 | extracellular matrix structural constituent | 6.3142E-05 | 5.087728612 |
| GO:0016702 | oxidoreductase activity, acting on single donors with incorporation of molecular oxygen, incorporation of two atoms of oxygen | 0.00075435 | 11.41912422 |
| GO:0016705 | oxidoreductase activity, acting on paired donors, with incorporation or reduction of molecular oxygen | 0.00080113 | 3.952773768 |
| GO:0001102 | RNA polymerase II activating transcription factor binding | 0.00123964 | 5.505649177 |
| GO:0042626 | ATPase activity, coupled to transmembrane movement of substances | 0.00152647 | 6.58795628 |
| GO:0004611 | phosphoenolpyruvate carboxykinase activity | 0.00214751 | 51.38605898 |
| GO:0004613 | phosphoenolpyruvate carboxykinase (GTP) activity | 0.00214751 | 51.38605898 |
| GO:0046983 | protein dimerization activity | 0.00245473 | 2.740589812 |
| GO:0051213 | dioxygenase activity | 0.00278017 | 3.996693476 |
| GO:0004409 | homoaconitate hydratase activity | 0.00353342 | 34.25737265 |
| GO:0044212 | transcription regulatory region DNA binding | 0.00510904 | 2.486422209 |
| GO:0043565 | sequence-specific DNA binding | 0.00551417 | 1.771933068 |
| GO:0003707 | steroid hormone receptor activity | 0.00606042 | 3.902738657 |
| GO:0004332 | fructose-bisphosphate aldolase activity | 0.00723216 | 20.55442359 |
| GO:0015276 | ligand-gated ion channel activity | 0.00724642 | 8.564343164 |
| GO:0003700 | sequence-specific DNA binding transcription factor activity | 0.00805009 | 1.581767207 |
| GO:0051393 | alpha-actinin binding | 0.00827148 | 8.11358826 |
| GO:0015105 | arsenite transmembrane transporter activity | 0.00952023 | 17.12868633 |
| GO:0030371 | translation repressor activity | 0.00952023 | 17.12868633 |
| GO:0016706 | oxidoreductase activity, acting on paired donors, with incorporation or reduction of molecular oxygen, 2-oxoglutarate as one donor, and incorporation of one atom each of oxygen into both donors | 0.01000527 | 5.138605898 |
| *CC - downregulation* | |  |  |
| GO:0005576 | extracellular region | 3.2728E-07 | 1.796951496 |
| GO:0005615 | extracellular space | 1.6091E-05 | 1.978528991 |
| GO:0000786 | nucleosome | 6.9894E-05 | 4.249023013 |
| GO:0005887 | integral component of plasma membrane | 0.00115135 | 1.570268272 |
| GO:0005921 | gap junction | 0.002143 | 4.261447057 |
| GO:0045202 | synapse | 0.00216613 | 1.860092946 |
| GO:0009897 | external side of plasma membrane | 0.00222436 | 2.45521377 |
| GO:0072562 | blood microparticle | 0.00455627 | 34.70035461 |
| GO:0030054 | cell junction | 0.00480296 | 1.584146623 |
| GO:0005577 | fibrinogen complex | 0.00640033 | 9.463733075 |
| GO:0045211 | postsynaptic membrane | 0.00645925 | 2.055421702 |
| GO:0043205 | fibril | 0.00745194 | 23.13356974 |
| GO:0060342 | photoreceptor inner segment membrane | 0.00783399 | 8.675088652 |
| GO:0031091 | platelet alpha granule | 0.00783399 | 8.675088652 |
| GO:0043025 | neuronal cell body | 0.00939744 | 1.927797478 |
| GO:0030016 | myofibril | 0.0096106 | 3.154577692 |
| GO:0005922 | connexon complex | 0.01012306 | 4.130994596 |
| GO:0016021 | integral component of membrane | 0.01223784 | 1.217445635 |
| GO:0005578 | proteinaceous extracellular matrix | 0.01571665 | 1.730896548 |
| GO:0032982 | myosin filament | 0.01970393 | 4.206103589 |
| CC - upregulation | |  |  |
| GO:0005604 | basement membrane | 1.9375E-06 | 5.641183122 |
| GO:0033391 | chromatoid body | 0.00015391 | 18.77764452 |
| GO:0005578 | proteinaceous extracellular matrix | 0.00047096 | 2.453136457 |
| GO:0005615 | extracellular space | 0.00060419 | 1.925120354 |
| GO:0016281 | eukaryotic translation initiation factor 4F complex | 0.00061539 | 12.15024057 |
| GO:0005887 | integral component of plasma membrane | 0.00090829 | 1.723837915 |
| GO:0005788 | endoplasmic reticulum lumen | 0.0009104 | 2.944301717 |
| GO:0016324 | apical plasma membrane | 0.00180819 | 2.600501129 |
| GO:0043186 | P granule | 0.00216272 | 14.08323339 |
| GO:0046658 | anchored component of plasma membrane | 0.0043487 | 6.663035152 |
| GO:0045211 | postsynaptic membrane | 0.00592026 | 2.339027148 |
| GO:0031012 | extracellular matrix | 0.00715442 | 2.204083274 |
| GO:0005581 | collagen trimer | 0.00969862 | 2.849021927 |
| GO:0005576 | extracellular region | 0.01131193 | 1.427912888 |
| GO:0005606 | laminin-1 complex | 0.01197304 | 14.75386355 |
| GO:0071546 | pi-body | 0.01197304 | 14.75386355 |
| GO:0005886 | plasma membrane | 0.0120661 | 1.288162707 |
| GO:0031090 | organelle membrane | 0.01343775 | 2.915077879 |
| GO:0030054 | cell junction | 0.01932199 | 1.571607204 |
| GO:0005577 | fibrinogen complex | 0.0246604 | 9.38882226 |

Table S5 Typical significant genes involving in glycolysis and OXPHOS.

| **Symbol** | **BLAST** | **Type** | **10C** | **FC (treatment/control)** | **24C** |
| --- | --- | --- | --- | --- | --- |
| *glycolysis (10H)* |  |  |  |  |  |
| pfkpb | PFKP | phosphofructokinase |  | 2.50 (147.161/58.757) | 66.321 |
| aldocb | ALDOC | fructose-bisphosphate aldolase |  | 12.56 (949.191/75.544) | 1989.627 |
| tpi1b | TPI1 | triosephosphate isomerase |  | 2.12 (5552.189/2623.069) | 8431.314 |
| gapdhs | GAPDHS | glyceraldehyde phosphate dehydrogenase |  | 76.23 (6878.744/90.234) | 5063.923 |
| pgk1 | PGK1 | phosphoglycerate kinase |  | 2.77 (1679.742/605.404) | 5561.33 |
| eno1a | ENO1 | enolase |  | 64.19 (2694.105/41.969) | 2644.279 |
| pkma | PKM | pyruvate kinase |  | 2.13 (483.530/226.633) | 1555.332 |
| ldha | LDHA | lactate dehydrogenase |  | 9.55 (4206.714/440.676) | 5870.471 |
| ldhbb | LDHB |  |  | 2.34 (58.865/25.181) | 44.927 |
| adpgk2 | ADPGK | ADP-dependent glucokinase |  | 2.3 (901.889/391.362) | 241.75 |
| gpia | GPI | glucose-6-phosphate isomerase |  | 3.25 (3074.622/946.403) | 2248.493 |
| gpib | GPI |  |  | 11.02 (11.563/1.049) | 536.985 |
| LOC564852 | PFKFB2 | fructose-2,6-biphosphatase |  | 2.08 (30.483/14.689) | 34.23 |
| pfkfb3 | PFKFB3 |  |  | 16.88 (3329.001/197.255) | 161.524 |
| pck1 | PCK1 | phosphoenolpyruvate carboxykinase |  | 5.59 (70.427/12.591) | 440.713 |
| LOC557008 | ALDH3B1 | aldehyde dehydrogenase |  | 3.26 (13.665/4.197) | 19.254 |
| *OXPHOS (24H)* | |  |  |  |  |
| ndufa10 | NDUFA10 | NADH-coenzyme Q oxidoreductase (complex I) | 5282.891 | 0.43 (3141.166/7264.280) |  |
| ndufa12 | NDUFA12 |  | 2277.873 | 0.42 (1557.225/3722.529) |  |
| ndufab1 | NDUFAB1 |  | 1795.228 | 0.39 (1040.161/2698.833) |  |
| ndufb1 | NDUFB1 |  | 318.965 | 0.38 (505.861/1316.791) |  |
| ndufb3 | NDUFB3 |  | 2082.717 | 0.49 (1627.891/3292.512) |  |
| ndufb5 | NDUFB5 |  | 1556.004 | 0.50 (1128.924/2261.329) |  |
| ndufb6 | NDUFB6 |  | 2468.832 | 0.40 (1589.111/3958.931) |  |
| ndufb8 | NDUFB8 |  | 4121.366 | 0.43 (3419.519/7923.210) |  |
| ndufb10 | NDUFB10 |  | 1279.008 | 0.38 (2013.104/5338.834) |  |
| LOC100000992 | NDUFC1 |  | 971.585 | 0.27 (949.675/3503.242) |  |
| ndufs4 | NDUFS4 |  | 1620.007 | 0.42 (1976.909/4698.088) |  |
| ndufs6 | NDUFS6 |  | 1975.695 | 0.47 (1942.438/4158.963) |  |
| ndufs7 | NDUFS7 |  | 2978.757 | 0.49 (2245.783/4580.422) |  |
| ndufs8a | NDUFS8 |  | 4387.87 | 0.41 (3405.730/8263.372) |  |
| sdhc | SDHC | Succinate-Q oxidoreductase (complex II) | 2215.969 | 0.45 (2699.938/5935.722) |  |
| sdhdb | SDHD |  | 970.535 | 0.35 (1549.469/4460.616) |  |
| LOC100333064 | UQCR10 | Q-cytochrome c oxidoreductase (complex III) | 4678.506 | 0.29 (3298.009/11441.427) |  |
| uqcr11 | UQCR11 |  | 4004.902 | 0.39 (2711.141/6944.442) |  |
| uqcrq | UQCRQ |  | 3928.308 | 0.36 (2962.779/8336.111) |  |
| cox5aa | COX5A | Cytochrome c oxidase (complex IV) | 3463.5 | 0.47 (2355.228/5009.368) |  |
| cox5ab | COX5A |  | 2264.233 | 0.45 (1949.333/4289.466) |  |
| cox7a2 | COX7A2 |  | 2956.723 | 0.25 (2917.966/11599.742) |  |
| cox7c | COX7C |  | 7160.978 | 0.26 (3608.247/13725.220) |  |
| cox11 | COX11 |  | 271.75 | 0.41 (367.977/889.984) |  |
| cox17 | COX17 |  | 1057.621 | 0.43 (1861.432/4349.368) |  |
| atp5c1 | ATP5C1 | ATP synthase (complex V) | 6141.129 | 0.35 (5603.254/16173.746) |  |
| atp5g3b | ATP5G3 |  | 20097.258 | 0.48 (13256.668/27734.979) |  |
| atp5h | ATP5H |  | 3973.425 | 0.30 (3545.338/11794.426) |  |
| atp5ia | ATP5I |  | 1122.673 | 0.18 (1315.929/7134.847) |  |
| atp5ib | ATP5I |  | 3654.46 | 0.49 (5373.160/11071.314) |  |
| atp5j | ATP5J |  | 4906.188 | 0.23 (2651.678/11449.985) |  |
| LOC402883 | ATP5J2 |  | 11288.66 | 0.27 (4607.043/17024.151) |  |
| atp5l | ATP5L |  | 4171.729 | 0.31 (2789.562/8951.184) |  |
| atp5o | ATP5O |  | 9653.943 | 0.48 (13351.463/27855.854) |  |

Table S6 Typical significant genes in the binding and signaling of retinoids.

| **GOTerm** | **10H** | **Style** | **24H** | **Style** |
| --- | --- | --- | --- | --- |
| retinol binding/retinal binding/retinoid binding/retinoic acid binding |  |  | crabp1a | down |
| retinol binding/retinal binding/retinoid binding/retinoic acid binding | crabp1b | down | crabp1b | down |
| retinol binding/retinal binding/retinoid binding/retinoic acid binding | crabp2a | down | crabp2a | down |
| retinol binding/retinal binding/retinoid binding | rbp5 | down | rbp5 | down |
| retinol binding/retinal binding/retinoid binding | rbp2a | down | rbp2a | down |
| retinol binding/retinal binding/retinoid binding | rbp2b | down | rbp2b | down |
| retinol binding/retinal binding/retinoid binding | rbp4 | down | rbp4 | down |
| retinol binding/retinal binding/retinoid binding | rbp4l | down |  |  |
| retinol binding/retinoic acid binding |  |  | lrata | down |
| retinol binding | rlbp1a | down |  |  |
| retinol binding |  |  | rlbp1b | down |
| retinol binding |  |  | c8g | down |
| retinal binding | opn1mw1 | down |  |  |
| retinal binding |  |  | exorh | down |
| retinoid binding |  |  | lcn15 | down |
| retinoic acid binding | cyp26b1 | down |  |  |
| retinoic acid binding | cyp26c1 | down |  |  |
| retinoic acid binding | nr2f2 | down |  |  |
| retinoic acid binding | nr2f5 | down |  |  |
| retinoic acid binding | ugt1a4 | down | ugt1a4 | down |
| retinoic acid binding |  |  | ugt1a1 | down |
| retinoic acid binding |  |  | ugt1a2 | down |
| retinoic acid binding |  |  | ugt1a5 | down |
| retinoic acid binding |  |  | ugt1ab | down |
| retinoic acid receptor activity/retinoic acid-responsive element binding |  |  | rarab | up |
| retinoic acid receptor activity/retinoic acid-responsive element binding |  |  | raraa | up |
| retinoic acid receptor activity/retinoic acid-responsive element binding |  |  | rxrab | up |
| retinoic acid receptor activity/retinoic acid-responsive element binding |  |  | rxrgb | up |
| retinoic acid receptor activity |  |  | esrrgb | up |
| retinoic acid receptor activity |  |  | rargb | up |
| retinoid X receptor binding |  |  | pparg | up |
| retinoid X receptor binding |  |  | nr1h4 | up |
| retinoid X receptor binding |  |  | nr1h5 | up |
| retinoid X receptor binding |  |  | nsd1b | up |

Table S7 Typical significant genes in three families of transmembrane transporters.

|  |  |  | Fold Change (treatment/control) | |  |
| --- | --- | --- | --- | --- | --- |
| Gene Symbol | Accession | Blast Symbol | 10H | 24L | 24H |
| *Solute carrier family* | |  |  |  |  |
| slc1a3b | NM_001190303 | SLC1A3/EAAT1 |  |  | 0.06 (15.512/239.611) |
| slc2a1a | NM_001039808 | SLC2A1/GLUT1 |  |  | 5.18 (3377.292/651.443) |
| slc2a15a | NM_001162541 | SLC2A9/GLUT9 |  |  | 0.10 (110.307/1140.292) |
| slc4a1a | NM_198338 | SLC4A1/AE1 | 0.19 (36.790/189.910) |  | 0.06 (961.740/16137.376) |
| LOC100150452 | XM_001922928 | SLC6A8 | 37.07 (233.356/6.295) |  |  |
| slc7a3a | NM_001007329 | SLC7A3/CAT3 |  |  | 7.74 (6451.239/833.290) |
| slc7a10a | XM_690716 | SLC7A10/ASC1 |  |  | 5.89 (635.990/108.039) |
| slc10a4 | NM_001044803 | SLC10A4/P4 |  |  | 0.11 (41.365/372.253) |
| slc16a9a | NM_200410 | SLC16A9/MCT9 | 20.17 (317.448/15.738) |  | 7.80 (450.708/57.763) |
| slc16a9b | NM_001003552 | SLC16A9/MCT9 |  |  | 5.43 (3457.437/636.467) |
| slc22a5 | XM_009295741 | SLC22A5/OCTN2 |  | 31.81 (510.449/16.045) | 60.80 (975.528/16.045) |
| slc22a7a | NM_001083861 | SLC22A7/OAT2 |  |  | 0.06 (103.413/1827.034) |
| slc24a5 | NM_001030280 | SLC24A5/NCKX5 |  |  | 0.08 (45.674/542.340) |
| slc25a25a | NM_213257 | SLC25A25/APC3 |  |  | 0.15 (469.667/3043.274) |
| slc25a38a | NM_001076602 | SLC25A38 |  |  | 9.59 (779.905/81.297) |
| slc25a4 | NM_214702 | SLC25A4/ANT1 |  |  | 0.004 (19.821/5044.668) |
| slc32a1 | NM_001080701 | SLC32A1/VGAT |  |  | 0.16 (72.389/439.644) |
| slc38a3 | NM_001002648 | SLC38A3/SNAT3 |  |  | 6.83 (21190.16/3103.177) |
| slc45a2 | NM_001110377 | SLC45A2/MATP |  |  | 0.19 (302.483/1602.399) |
| LOC556497 | XM_679321 | SLC46A3 |  |  | 0.19 (43.950/231.054) |
| LOC555258 | XM_677725 | SLC47A1/MATE1 |  | 9.21 (177.368/19.254) | 32.63 (628.233/19.254) |
| *Ion channels (cation)* | |  |  |  |  |
| cacna1fb | XP_002663165 | CACNA1F/Cav1.4 | 2.30 (4346.517/1886.511) | | 0.36 (1712.345/4703.436) |
| cacna1sa | NM_001146150 | CACNA1S/Cav1.1 |  |  | 0.09 (31.024/327.326) |
| cacnb4b | NM_001113516 | CACNB4 |  |  | 0.39 (340.401/881.426) |
| kcnip1b | NM_001008632 | KCNIP1 |  |  | 0.43 (138.746/319.838) |
| kcnj11 | NM_001039827 | KCNJ11/Kir6.2 |  |  | 0.43 (154.258/360.486) |
| kcnj13 | NM_001045549 | KCNJ13/Kir7.1 | 2.87 (798.876/278.045) |  |  |
| kcnk1a | NM_001098753 | KCNK1/K2p1.1 |  |  | 2.46 (539.470/219.287) |
| kcnk5b | NM_200633 | KCNK5/K2p5.1 |  |  | 2.20 (620.477/282.399) |
| scn1ba | NM_001077539 | SCN1B/Navβ1 |  |  | 0.35 (210.273/608.655) |
| scn8ab | NM_001045183 | SCN8A/Nav1.6 |  |  | 0.42 (92.210/219.287) |
| *ATP-binding cassette transporters* | | |  |  |  |
| abca1b | NM_001114586 | ABCA1/ABC1 |  |  | 2.11 (23061.07/10939.74) |
| abca5 | NM_001099246 | ABCA5/ABC13 |  |  | 2.11 (1117.721/529.498) |
| abca12 | XM_681540 | ABCA12 |  | 2.35 (5458.196/2318.023) | 3.15 (7310.428/2318.023) |
| abcb5 | XM_001922682 | ABCB1/PGP |  |  | 4.10 (5311.974/1294.328) |
| abcb6a | NM_001145693 | ABCB6/PRP | 2.16 (3019.962/1396.522) | |  |
| abcb10 | XM_001343182 | ABCB10/M-ABC2 |  |  | 0.45 (500.691/1112.480) |
| abcb11a | XM_009304974 | ABCB11/BSEP | 0.42 (1389.624/3297.722) | 6.21 (2359.409/379.741) | 27.21 (10334.39/379.741) |
| LOC100002917 | XM_003198146 | ABCC1/MRP1 | 2.16 (872.457/403.953) | 3.67 (733.191/200.032) | 3.61 (723.028/200.032) |
| LOC100334180 | XM_009299629 | ABCC1/MRP1 |  |  | 7.86 (344.710/43.857) |
| abcc2 | NM_200589 | ABCC2/MRP2 |  | 2.80 (4619.822/1652.674) | 5.73 (9466.586/1652.674) |
| LOC795669 | XP_001335921 | ABCC3/MRP3 | 2.99 (1630.338/544.549) |  |  |
| abcd3a | NM_213482 | ABCD3/PMP70 | 0.48 (270.146/567.632) |  |  |
| abcf2a | NM_201315 | ABCF2 |  |  | 0.39 (1774.392/4596.467) |
| abcg2a | NM_001042775 | ABCG2/ABCP | 2.65 (228.100/86.037) |  | 2.37 (413.652/174.360) |

Table S8 Typical significant genes involving in somitogenesis.

| **Symbol** | **Aliase** | **FC (24H/24C)** | **10C** | **Symbol** | **Aliase** | **FC (24H/24C)** | |  | **10C** |
| --- | --- | --- | --- | --- | --- | --- | --- | --- | --- |
| aldoaa |  | 4.09 (73887.630/18049.986) | 42934.39 | itga5 |  | 2.60 (2451.747/943.468) | | | 1822.508 |
| cdh2 |  | 2.05 (16297.006/7957.440) | 23518.44 | lef1 |  | 2.49 (1633.061/656.791) | | | 2590.543 |
| cdx1a |  | 3.82 (543.779/142.269) | 10543.69 | mespaa |  | 99.29 (424.855/4.279) | | | 221.387 |
| cdx4 |  | 36.09 (5096.531/141.199) | 26787.83 | mespab |  | 208.25 (445.537/2.139) | | | 561.337 |
| chd |  | 3.94 (370.563/94.133) | 13595.89 | mespbb |  | 218.33 (233.541/1.070) | | | 333.654 |
| cyp26a1 |  | 4.25 (5521.385/1298.606) | 8436.839 | msgn1 |  | 80.36 (6016.905/74.878) | | | 20858.64 |
| efnb2a |  | 2.64 (1671.841/632.188) | 932.763 | myf5 |  | 12.51 (3505.696/280.259) | | | 3953.489 |
| fgf8a |  | 5.63 (2283.701/405.413) | 4629.192 | neo1a |  | 2.18 (5894.533/2707.391) | | | 1323.076 |
| fn1a |  | 2.94 (9487.269/3229.401) | 41676.37 | pcdh8 |  | 52.46 (2917.966/55.624) | | | 3326.051 |
| fn1b |  | 12.23 (29233.955/2390.762) | 11413.5 | psen2 |  | 2.37 (2676.670/1127.456) | | | 1358.75 |
| foxb1a | foxb1.2 | 3.77 (1290.937/342.301) | 1617.909 | pxna |  | 3.20 (815.238/254.587) | | | 402.903 |
| fxr1 |  | 2.81 (6865.752/2443.177) | 7707.626 | ripply1 |  | 12.59 (2573.257/204.311) | | | 2824.521 |
| gadd45ba | | 2.72 (1083.250/397.925) | 924.369 | ta | ntla | 3.43 (1990.698/579.773) | | | 5223.055 |
| gadd45bb | | 3.44 (720.443/209.660) | 1079.655 | tbx6 | tbx24 | 64.34 (4335.584/67.391) | | | 8716.982 |
| gli2a |  | 2.75 (6873.508/2502.010) | 2999.742 | tbx16 |  | 85.61 (2381.081/27.812) | | | 5298.599 |
| has2 |  | 11.82 (860.050/72.739) | 743.902 | tcf15 | par1 | 5.05 (2219.930/439.643) | | | 2196.033 |
| her1 |  | 137.44 (735.093/5.348) | 1759.555 | tdgf1 | oep | 7.94 (1028.096/129.433) | | | 1100.64 |
| her7 |  | 42.86 (458.464/10.697) | 2194.984 | wnt3a |  | 2.32 (240.435/103.760) | | | 109.12 |
| igf2b |  | 2.39 (2238.889/938.120) | 150.04 |  |  |  |  |  |  |

Table S9 Typical significant genes involving in lens structure.

| **Symbol** | **Description** | **FC (24H/24C)** | **Symbol** | **Description** | **FC (24H/24C)** |
| --- | --- | --- | --- | --- | --- |
| crygm2d1 | crystallin, gamma M2d1 | 0.0008 (1.724/2282.723) | cryaa | crystallin, alpha A | 0.05 (10.341/189.336) |
| crygm2d2 | crystallin, gamma M2d2 | 0.002 (1.724/801.199) | cryba1b | crystallin, beta A1b | 0.02 (117.201/5913.258) |
| crygm2d3 | crystallin, gamma M2d3 | 0 (0/2347.974) | cryba1l1 | crystallin, beta A1, like 1 | 0.03 (208.549/6773.291) |
| crygm2d4 | crystallin, gamma M2d4 | 0.002 (5.171/2660.324) | cryba2a | crystallin, beta A2a | 0.03 (115.478/4593.258) |
| crygm2d5 | crystallin, gamma M2d5 | 0 (0.862/1584.214) | cryba2b | crystallin, beta A2b | 0.02 (168.908/7124.150) |
| crygm2d6 | crystallin, gamma M2d6 | 0 (0/434.295) | cryba4 | crystallin, beta A4 | 0.01 (44.812/3125.641) |
| crygm2d7 | crystallin, gamma M2d7 | 0.005 (5.171/1098.574) | crybb1 | crystallin, beta B1 | 0.02 (119.787/5486.451) |
| crygm2d8 | crystallin, gamma M2d8 | 0.004 (21.544/5626.581) | crybb1l1 | crystallin, beta B1, like 1 | 0.01 (76.698/5397.667) |
| crygm2d9 | crystallin, gamma M2d9 | 0.001 (3.447/2868.914) | crybb1l2 | crystallin, beta B1, like 2 | 0.02 (187.005/7725.317) |
| crygm2d10 | crystallin, gamma M2d10 | 0.003 (17.235/5796.662) | crygn2 | crystallin, gamma N2 | 0.02 (82.730/5294.976) |
| crygm2d12 | crystallin, gamma M2d12 | 0.003 (5.171/1755.365) | cryl1 | crystallin, lambda 1 | 0.44 (544.641/1236.564) |
| crygm2d13 | crystallin, gamma M2d13 | 0.002 (5.171/2212.123) | crym | crystallin, mu | 0.46 (85.316/183.987) |
| crygm2d15 | crystallin, gamma M2d15 | 0 (0/193.614) | hspb9 | heat shock protein, alpha-crystallin-related, 9 | 0.30 (174.940/590.470) |
| crygm2d16 | crystallin, gamma M2d16 | 0 (0/284.538) | mipa | major intrinsic protein of lens fiber a | 0.009 (25.853/2914.911) |
| crygm2d19 | crystallin, gamma M2d19 | 0.003 (15.512/5363.437) | mipb | major intrinsic protein of lens fiber b | 0.007 (11.203/1607.747) |
| crygm2d21 | crystallin, gamma M2d21 | 0.002 (6.032/3064.668) |  |  |  |
| crygmx | crystallin, gamma MX | 0.01 (5.171/398.995) |  |  |  |
| crygmxl2 | crystallin, gamma MX, like 2 | 0.004 (5.171/1405.576) |  |  |  |
